# Sodium Ascorbate‐Accelerated Gelling Hydrogels With Rapid Self‐Mineralized Capacity for Chronic Wounds

**DOI:** 10.1002/advs.75881

**Published:** 2026-05-29

**Authors:** Xiaoya Ding, Wei Yang, Wenzhao Li, Guifang Xu, Yuanjin Zhao

**Affiliations:** ^1^ MOE Innovation Center For Basic Research in Tumor Immunotherapy Anhui Province Key Laboratory of Tumor Immune Microenvironment and Immunotherapy The First Affiliated Hospital of Anhui Medical University Hefei China; ^2^ Department of Rheumatology and Immunology Nanjing Drum Tower Hospital School of Biological Science and Medical Engineering Southeast University Nanjing China; ^3^ Department of Gastroenterology The First Affiliated Hospital of Anhui Medical University Hefei China

**Keywords:** immunoregulatory, SA‐accelerated gelling hydrogels, self‐mineralization, wound healing

## Abstract

Chronic wound management remains clinically challenging owing to the persistent inflammation, excessive reactive oxygen species (ROS), potential bacterial infections, and the complex immune microenvironment. To address these issues, we develop a novel immunoregulatory hydrogel platform based on sodium ascorbate (SA)‐accelerated gelation and in situ self‐mineralization for facilitating the infected chronic diabetic wound healing. Such a hydrogel is constructed by mixing benzaldehyde‐ and cyanoacetate‐functionalized dextran with silver nitrate (AgNO_3_) and SA, which enables not only the ultrafast solidification but also in situ self‐mineralization of silver nanoparticles (Ag NPs). The accelerated gelling properties and ROS‐scavenging of the incorporated SA aid in rapid wound closure and oxidative stress alleviation, while the in situ‐generated Ag NPs embedded within the hydrogel matrix provide multimodal antibacterial performance, effectively ameliorating inflammatory responses. Thus, when used in an infected diabetic wound model, this immunoregulatory hydrogel platform demonstrates significant facilitation effects for the wound healing by promoting wound closure, alleviating oxidative stress, eradicating bacterial infection, and inhibiting inflammation. These integrated features endow the hydrogels with high value for clinical wound management.

## Graphical abstract

An immunoregulatory hydrogel based on sodium ascorbate (SA)‐accelerated gelation and in situ self‐mineralization is prepared here. Such a hydrogel is constructed by mixing benzaldehyde‐ and cyanoacetate‐functionalized dextran with silver nitrate (AgNO_3_) and SA, enabling the ultrafast solidification and in situ self‐mineralization of silver nanoparticles (Ag NPs). The resulting hydrogel exhibited rapid wound closure, alleviation of oxidative stress, and multimodal antibacterial activity, thereby effectively promoting infected chronic wound healing through enhanced epithelialization, oxidative stress relief, bacterial eradication, and inflammation inhibition.

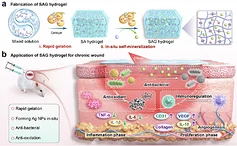

## 1 Introduction

The management of chronic refractory wounds following trauma or surgical procedures poses significant challenges in the clinic, imposing substantial social and economic burdens worldwide and severely compromising patients’ quality of life [1, 2]. Evidence indicates that persistent inflammation, caused by pathogen infection and overproduced reactive oxygen species (ROS), represents a critical barrier to the healing of chronic wounds for patients with diabetes [3, 4, 5]. Particularly, wounds exposed to a local hyperglycemic environment have an increased incidence of pathogenic infection because of the sufficient nutrition and compromised skin barrier, which then triggers pro‐inflammatory cytokine release from immune cells and ultimately drives persistent inflammatory responses [6, 7, 8, 9]. Moreover, the excessive ROS produced by immune cells (e.g., macrophages and neutrophils) inflicts irreversible damage on endogenous stem cells/biomolecules in the wounded tissue while maintaining macrophages in the pro‐inflammatory M1 phenotype to intensify the inflammatory response [10, 11, 12]. To combat these issues, strategies integrated with antibiotics or antioxidants have been extensively proposed [13, 14, 15]. Despite much progress, employing antibiotics against wound infections poses a significant risk to induce bacterial resistance, leading to the limited antibacterial effect and diminished therapeutic efficacy [16, 17, 18, 19]. Additionally, owing to the structural and functional constraint of antioxidants or limited scavenging efficacy, the current therapeutic strategies can only partially eliminate the specific ROS species, thereby failing to achieve sufficient inflammation amelioration. Therefore, developing an all‐rounded therapeutic platform with the abilities of rapid wound closure, antibacterial, ROS scavenging, and regulation of inflammatory responses is urgently needed for effective management of chronic diabetic wounds.

**FIGURE 1:**
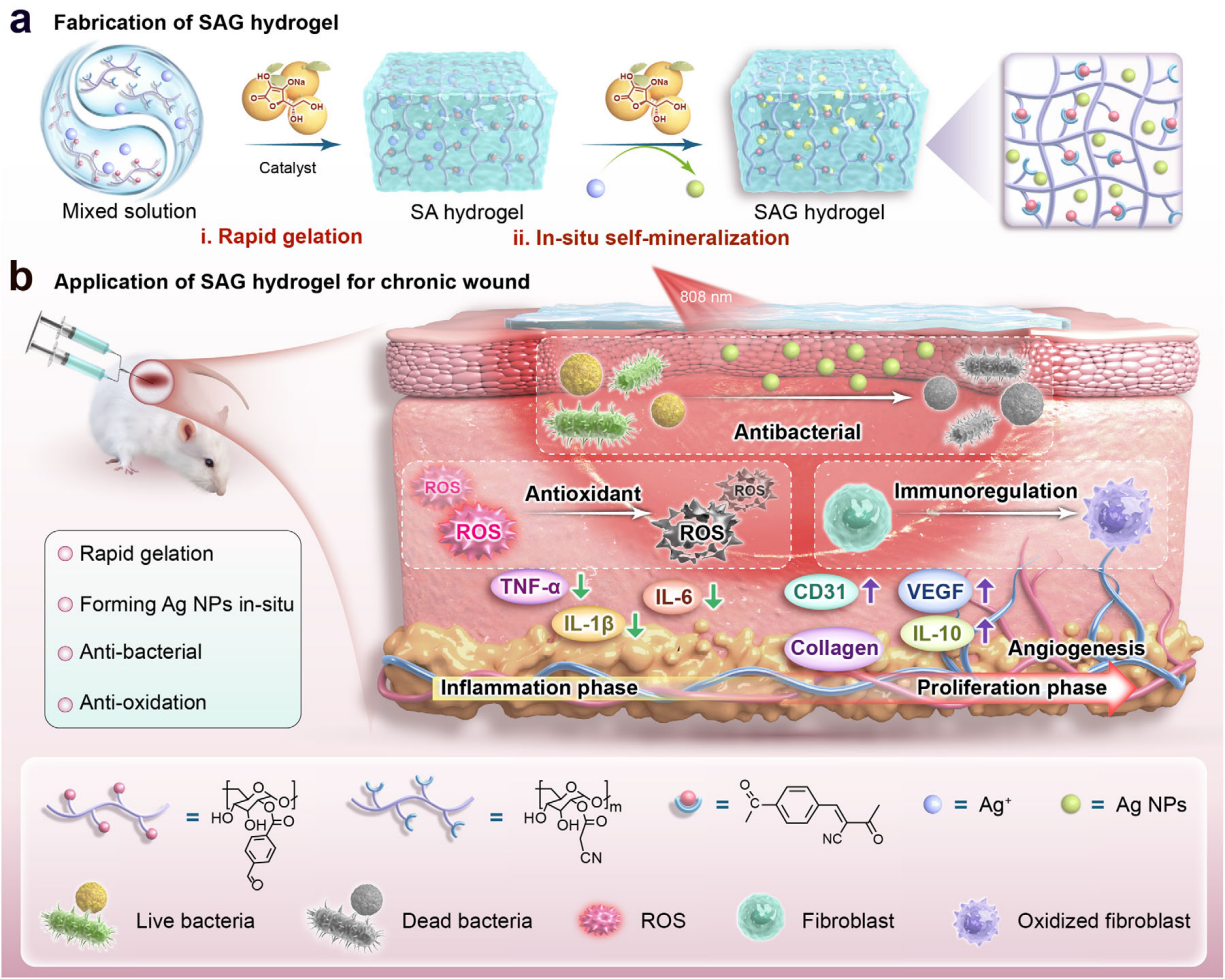
Schematic illustration of the SA‐accelerated gelling hydrogels with self‐mineralization for managing infected chronic diabetic wounds. (a) Preparation process of the immunoregulatory hydrogels. The hydrogel can be rapidly formed when mixed the solution composed of benzaldehyde‐ and cyanoacetate‐modified dextran and AgNO_3_ with the presence of SA, during which the rapid gelation occurs with the in situ reduction of Ag ions to form Ag NPs within the network. (b) The therapeutic mechanism of self‐mineralized hydrogel dressings. The multifunctional hydrogel accelerates wound healing by rapidly sealing the wound, scavenging excessive ROS, eradicating bacteria, and modulating the immune microenvironment.

Herein, we present a novel sodium ascorbate (SA)‐accelerated gelling hydrogel platform that enables rapid self‐mineralization of silver nanoparticles (Ag NPs) for the treatment of infected diabetic wounds, as schemed in Figure 1. Hydrogels, composed of hydrophilic polymers that form 3D networks to absorb and retain abundant water or biological fluids, have been considered as advanced wound dressings [20, 21, 22]. Notably, a variety of multifunctional hydrogels for wound repair have been designed that incorporates antibiotics or photothermal agents for antibacterial [23, 24, 25], polyphenolic substances or nanozymes for ROS scavenging [26, 27], and macrophage polarity‐modulating molecules for regulating the immune microenvironment [28, 29]. Nevertheless, these systems often suffer from tedious fabrication processes, poorly controlled gelation kinetics, low synergistic efficiency, and insufficient spatiotemporal precision. Specifically, the majority of previously reported Ag NPs‐based antibacterial hydrogel systems primarily rely on the pre‐synthesized nanoparticles, which results in a lack of the integrated antioxidant capacity required for effective modulation of the chronic wound microenvironment [30, 31, 32, 33]. Thus, there is an urgent requirement to develop a hydrogel dressing with simple compositions yet comprehensive therapeutic efficacy. SA, a natural antioxidant and recognized free‐radical scavenger, is extensively employed as skin products for photoprotection and anti‐pigmentation [34, 35]. Also, in other cases, SA can be employed as the reducing agent/surfactant in the synthesis of nanoparticles [36, 37]. While SA‐incorporated hydrogels demonstrated improved antioxidant capacity, their inherent antibacterial performance remains limited [38, 39]. In contrast, Ag NPs demonstrate broad and potent antibacterial properties, yet their practical application is often hampered by the aggregation‐prone synthesis and instability under physiological conditions [40, 41, 42]. Conventional methods for Ag NPs production, such as chemical reduction, laser ablation, and mechanical grinding, typically require the elevated temperature/pressure or toxic reagents [43, 44]. Conversely, biomineralization represents a promising alternative, offering a low‐energy and eco‐friendly approach for preparing nanoparticles that contributes to the uniform in situ formation and distribution within a polymeric matrix [45, 46]. Therefore, it is conceived that integrating SA and a silver precursor into one hydrogel system enables the facile fabrication of an immunomodulatory hydrogel for effective chronic wound management through in situ self‐mineralization of Ag NPs.

In this research, we developed a desired hydrogel with a dynamic C = C double bond‐crosslinked network and integrated immunoregulatory function for accelerated infected diabetic wound healing through the rapid wound closure, potent antioxidative ability, and multimodal antibacterial function. The hydrogel was readily formed upon mixing the precursor solution composed of benzaldehyde‐ and cyanoacetate‐modified dextran with Ag^+^ and SA, of which SA served as a gelation accelerator and a Ag^+^ mineralizer, respectively, enabling the ultrafast hydrogel formation and in situ self‐mineralization of Ag NPs. The embedded SA endowed the hydrogel with robust ROS‐scavenging capability, leading to the effective reduction of intracellular oxidative stress. Meanwhile, the uniformly distributed Ag NPs within the hydrogel matrix demonstrated notable photothermal conversion efficiency, contributing to both intrinsic and photothermal antibacterial capacities. Besides, the hydrogel also displayed favorable moldability and mechanical flexibility, allowing it to conform to irregular wounds. Benefitting from these combined features, the hydrogel dressing can significantly promote the repair of infected full‐thickness diabetic wounds by accelerating wound closure, alleviating oxidative damage, eradicating bacteria, and regulating inflammatory responses. These results imply that this hydrogel not only serves as a promising therapeutic platform for chronic wound management but also as a versatile strategy for treating other inflammation‐related diseases.

## 2 Results and Discussion

**FIGURE 2:**
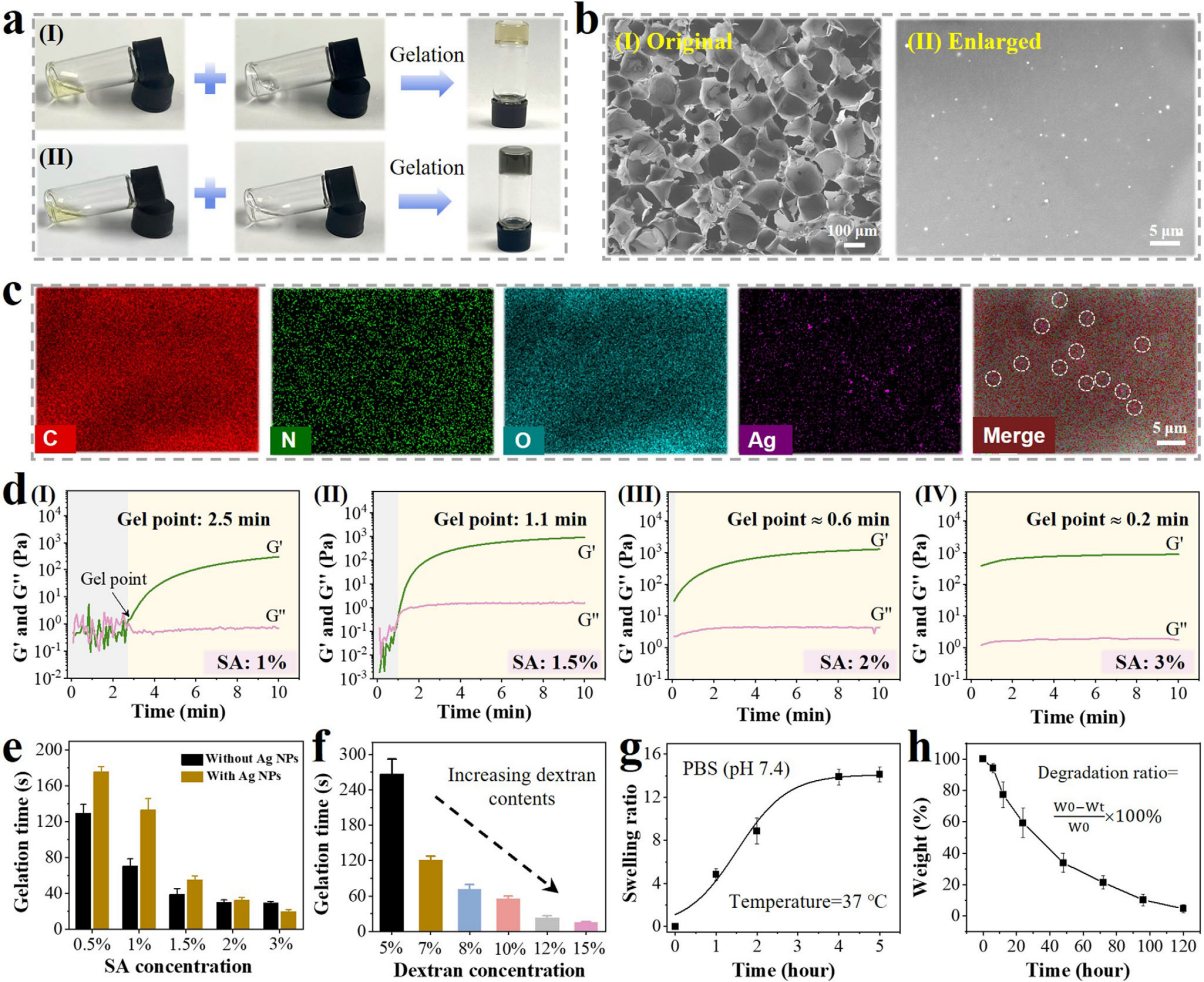
Preparation and characterization of the SAG hydrogel. (a) Pictures of sol–gel transition for hydrogels in the presence of SA (I) or two reagents of SA and AgNO_3_ (II). (b) The SEM images of SAG hydrogels and the enlarged SEM image of the formed Ag NPs within hydrogels. (c) Elemental mapping results of the SAG hydrogel. (d) For hydrogels with different contents of SA, G′ and G′′ were recorded with time. In particular, the gelation occurred too quickly to be measured precisely, so the approximate values were indicated for hydrogels prepared with 2% and 3% SA. (e) Gelation time of 10% SAG hydrogels in the presence or absence of different contents of SA at a fixed AgNO_3_ concentration of 0.01%. (f) Gelation time of SAG hydrogels with different dextran polymer contents with a fixed SA content of 1.5% and AgNO_3_ of 0.01%. (g) Swelling ratio of 10% SAG hydrogels. (h) Degradation curve of 10% SAG hydrogels.

In this work, the benzaldehyde and cyanoacetate group‐modified polymers were initially prepared via esterification reaction. By mixing the two polymers, the hydrogels based on benzaldehyde and cyanoacetate group‐modified polymers can be readily synthesized through the generation of C = C double bonds with the gelation time of about 5 min. Intriguingly, this gelation can be significantly accelerated when sodium ascorbate (SA) is introduced into the hydrogel precursor solution, specifically behaving as the remarkably shortened gelation time of about a few seconds (Figure 2a). Furthermore, such promotion effect was maintained even when silver nitrate (AgNO_3_) was added into the SA‐incorporated hydrogel precursor solutions, and the gelation time remained roughly several seconds, despite the noticeable hydrogel color change from pale yellow to brown (Figure S1). Based on these phenomena, we propose that SA acts as a catalyst to facilitate C = C bond formation. Furthermore, it serves as a reducing agent, enabling the in situ formation of silver nanoparticles (Ag NPs) via a rapid redox reaction with AgNO_3_. To verify this hypothesis, scanning electron microscopy (SEM) analysis was first performed in Figure 2b. The result showed that the SA‐incorporated hydrogel with AgNO_3_ (named SAG hydrogel) exhibited a porous structure with the scattered Ag NPs. Besides, the elements containing C, O, and N, derived from the hydrogel building blocks were uniformly distributed within the SAG hydrogel from the elemental mapping images, whereas the Ag element mainly existed in the way of the Ag clusters, in accordance with the formed Ag nanoparticles in SEM images (Figure 2c). The detailed contents of different elements were presented in Figure S2, and particularly inductively coupled plasma mass spectrometry (ICP‑MS) analysis revealed that the loading content of Ag NPs was about 1.04 µg/mL. Besides, the average diameter of the formed Ag nanoparticles was about 220 nm from the dynamic light scattering (DLS) results (Figure S3). The UV–vis absorption spectra displayed that the incorporation of Ag^+^ led to an apparent absorption peak around 400 nm that can be attributed to surface plasmon resonance of colloidal silver, which further indicated that Ag in various Ag─SA complexes exists in the form of nanoparticles (Figure S4). Whereafter, to deeply investigate the catalytic behavior of SA in the formation of SAG hydrogels, the rheological experiment was performed (Figure 2d). Storage modulus (G') and loss modulus (G'') with time were measured to demonstrate sol–gel transition process. For the 10% SAG hydrogel with 1% SA content, the gelation time was approximately 2.5 min from the crossover point where the G' exceeded G''. Besides, with increasing the SA concentrations, the gelation time can be significantly reduced, indicating that the gelation behavior can be accelerated by SA. In addition, the G' of hydrogels was largely independent of the SA concentrations, revealing that SA acts as the catalyst for the C = C double bond formation.

Furthermore, to demonstrate the effect of AgNO_3_ on the gelation, the inverted vial test was carried out (Figure 2e). The gelation time of the 10% hydrogel without Ag NPs was dramatically decreased with increasing SA concentrations. Particularly, for the 10% hydrogel with 2% and 3% SA, gelation occurred in less than 40 s. When AgNO_3_ was incorporated within the mixed dextran solution, the gelation time may be slightly prolonged owing to the partial consumption of SA by AgNO_3_ through the redox reaction. Nevertheless, they were all controlled within 2 min as SA contents ranging from 0.5% to 3%, and there was no obvious difference in gelation time for 10% hydrogel with 2% SA and 3% SA in the presence or absence of Ag NPs. Furthermore, for the hydrogels with 1.5% SA and 0.01% AgNO_3_, their gelation time can also be adjusted by changing dextran concentrations. With the dextran contents increasing from 5% to 15%, their gelation time showed a significantly reduction trend (Figure 2f). Especially, the gelation time can be reduced to 2 min for hydrogels generated from more than 8% dextran contents. The in situ gelation behavior of polymers was also evaluated (Figure S5). Spraying the mixed dextran solution with the incorporation of AgNO_3_ onto glasses and gloves soaked of SA can result in the rapid gelation, demonstrated the effective wound closure ability of the SAG hydrogel. Additionally, swelling and degradation behaviors were critical properties for hydrogels used in vivo. Therefore, the swelling and degradation test was carried out by immersing the hydrogel into PBS (pH 7.4) buffer solution. As shown in Figure 2g,h, the hydrogel exhibited good swelling performance and the swelling equilibrium can be reached after a few hours. Moreover, the SAG hydrogel degraded almost completely within 5 days in vitro. This degradation rate is considered beneficial for controlled in vivo scaffold degradation and the drug release. Based on this, the release performance of Ag NPs from the SAG hydrogel was further investigated (Figure S6). No burst release of Ag NPs was observed, and the release rate was largely consistent with the hydrogel degradation, demonstrating the sustained release behavior. These results demonstrated that the hydrogel is well‐matched to the typical wound healing timeline, potentially accelerating the process.

**FIGURE 3:**
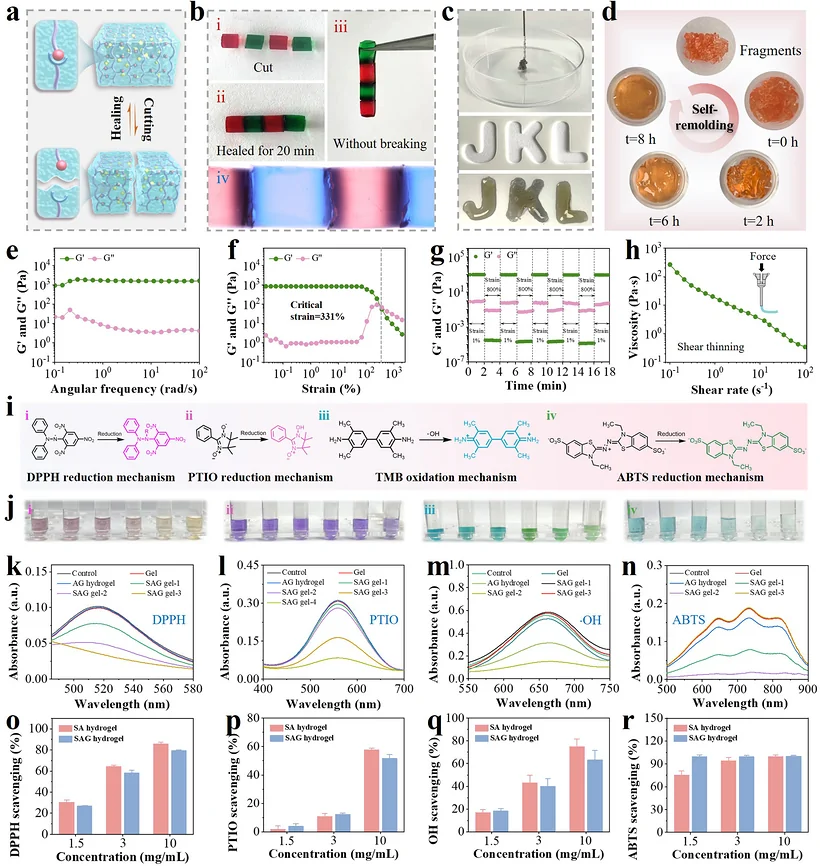
(a) Schematic diagram illustrating the self‐healing mechanism for SAG hydrogels. (b) Four separated SAG hydrogel blocks autonomously fused into an intact hydrogel when contacted due to the rapid self‐healing performance. (c) Injection of SAG hydrogels to generate hydrogel letter patterns. (d) Self‐remolding property of the SAG hydrogel. (e) Angular frequency sweep test of the SAG hydrogel. (f) Strain sweep test of the SAG hydrogel. (g) The step‐strain sweep of the SAG hydrogel when the applied strain switched between 1% and 800% at 37°C. (h) The viscosity of SAG hydrogel as a function of shear rate at 37°C. (i) Mechanisms of SAG hydrogel to scavenge the free radicals of DPPH, PTIO, ·OH, and ABTS, respectively. (j) The color changes of SAG hydrogel catalyzed by (i) DPPH free radicals, (ii) PTIO free radicals, (iii) ·OH, and (iv) ABTS. (k–n) UV–vis spectra of DPPH, PTIO, ·OH, and ABTS solution after treatment with different SAG hydrogels. (o–r) Quantitative scavenging capabilities for the various free radicals of DPPH, PTIO, ·OH, and ABTS treated by the different hydrogels.

Compared to the covalently crosslinked hydrogels, dynamic hydrogels based on the reversible covalent bonds are more attractive candidates to mimic the extracellular matrix (ECM) owing to their typical properties, such as self‐healing, remoldability, and injectability ability. In this work, the SAG hydrogels constructed by the reversible C = C double bonds were expected to possess excellent dynamic features owing to the enhanced dynamic exchange with the presence of the SA catalyst, which contributed to the rapid self‐healing performance (Figure 3a). To further confirm this, a macroscopic self‐healing assay was carried out. Four cylinder‐shaped hydrogel blocks stained red and green were separated and placed together for about 20 min. The repaired hydrogel could withstand its own weight without any damage and no crack was observed at the interface (Figure 3b). In addition to self‐healing, such hydrogels also exhibited the shear‐thinning performance, which was visually demonstrated via injecting the SAG hydrogel through a thin needle with no clogging. Due to the superior self‐healing ability of SAG hydrogel, the injected hydrogel could be molded into hydrogel letters of “J”, “K”, and “L” by employing the customized letter patterns (Figure 3c). Furthermore, the remoldability of SAG hydrogel was also investigated (Figure 3d). The hydrogel fragments could be fused into a complete hydrogel block without any cracks after incubation at room temperature for 8 h, which can be ascribed to the improved self‐healing capacity through the addition of SA catalyst.

Next, the rheological characterization of the SAG hydrogel was conducted to evaluate its mechanical properties. Frequency sweep result revealed a viscoelastic performance of the SAG hydrogel with the G′ consistently exceeding G'' across the entire frequency range, demonstrating the elastic solid‐like character (Figure 3e). Whereafter, a strain sweep test was conducted for determining the critical breakage strain (Figure 3f). The G′ and G′′ remained constant under a lower strain, suggesting its good anti‐shear ability, whereas G′ started to decrease at a strain of 100% and became lower than G′′ at about 331% strain, suggestive of the collapse of SAG hydrogel network. Next, the self‐healing behavior of the SAG hydrogel was further assessed by the continuous step–strain sweep test (Figure 3g). The hydrogel network collapsed under a high strain of 800% with the G′ lower than G′′, while the mechanical strength quickly recovered to the initial state once the low strain of 1% was applied. Besides, this disruption–recovery process can be repeated multiple times without significant G′ and G′′ loss, confirming the efficient self‐healing capacity of SAG hydrogels. Furthermore, viscosity variation of the SAG hydrogel was evaluated by exerting the changed shear rates, and the viscosity exhibited an obvious decreased trend with increasing the shear rate (Figure 3h), which again demonstrated the shear‐thinning property of SAG hydrogels.

Sodium ascorbate, a water‐soluble ketolactone, displayed the capacity to reduce radicals, such as hydroxyl radical, superoxide, and peroxyl radical [35]. Thus, it is proposed that the formed SAG hydrogel can scavenge the excessive free radicals under mild conditions. DPPH, a typical nitrogen‐free radical, has been employed as the model molecule to evaluate RNS eliminating capacity. ·OH and PTIO, the representative oxygen free radicals, are frequently used to assess ROS scavenging capacity. ABTS was a representative radical cationic that can also be applied to evaluate the antioxidant ability. Figure 3i displays the detailed radical scavenging mechanism of different radicals toward SAG hydrogels. First, the DPPH radical scavenging ability was evaluated in Figure 3j (i),k,o. The hydrogel without SA (named AG hydrogel) showed an insignificant effect on the absorption intensity as compared to the control group, whereas the absorption peak at 517 nm showed an obvious decrease for SAG hydrogels compared to the AG hydrogel. Besides, with increasing the SAG hydrogel contents, the absorption intensity at 517 nm became weaker and weaker, meanwhile the DPPH solution also became yellow (Figure 3j (i),k). In particular, the DPPH scavenging efficiency can reach up to approximately 80% as the SAG hydrogel concentration was raised to 10 mg/mL (Figure 3o). Next, the PTIO scavenging test was performed to assess the ability to remove oxygen‐free radicals (Figure 3j (ii),l,p). The SAG hydrogel exhibited stronger PTIO scavenging capacity than the other hydrogels from the results of the observed lighter color after treatment with SAG hydrogels. In addition, the absorbance at 560 nm showed an obvious decreased trend with increasing the SAG hydrogel contents, and when the SAG hydrogel contents were increased up to 10 mg/mL, nearly 60% PTIO can be eliminated. Furthermore, the ·OH scavenging capacity was investigated via using TMB solution as indicator (Figure 3j (iii),m,q). Similarly, the SAG hydrogels displayed the scavenging ability against ·OH as well, with the color of TMB substrate solution shifting to green, and the removal efficiency exhibited an increased trend with raising the SAG hydrogel amounts. Notably, when the SAG hydrogel was exposed to the ABTS solution, the color of ABTS solution became from azure to colorless, and the scavenging ratio can reach over 90%, demonstrating the excellent ABTS‐scavenging ability of SAG hydrogels (Figure 3j (iv),n,r). A similar case is that there are slight differences about the scavenging ratio between the SA and SAG hydrogels, indicating that the addition of AgNO_3_ had a minor effect on the antioxidant capacity. Together, these results indicated that the SAG hydrogel exhibited outstanding anti‐oxidation performance and had the ability to clear ROS and RNS at mild conditions, which would be beneficial for retaining the balance in the antioxidant system, thereby preventing oxidative damage.

**FIGURE 4:**
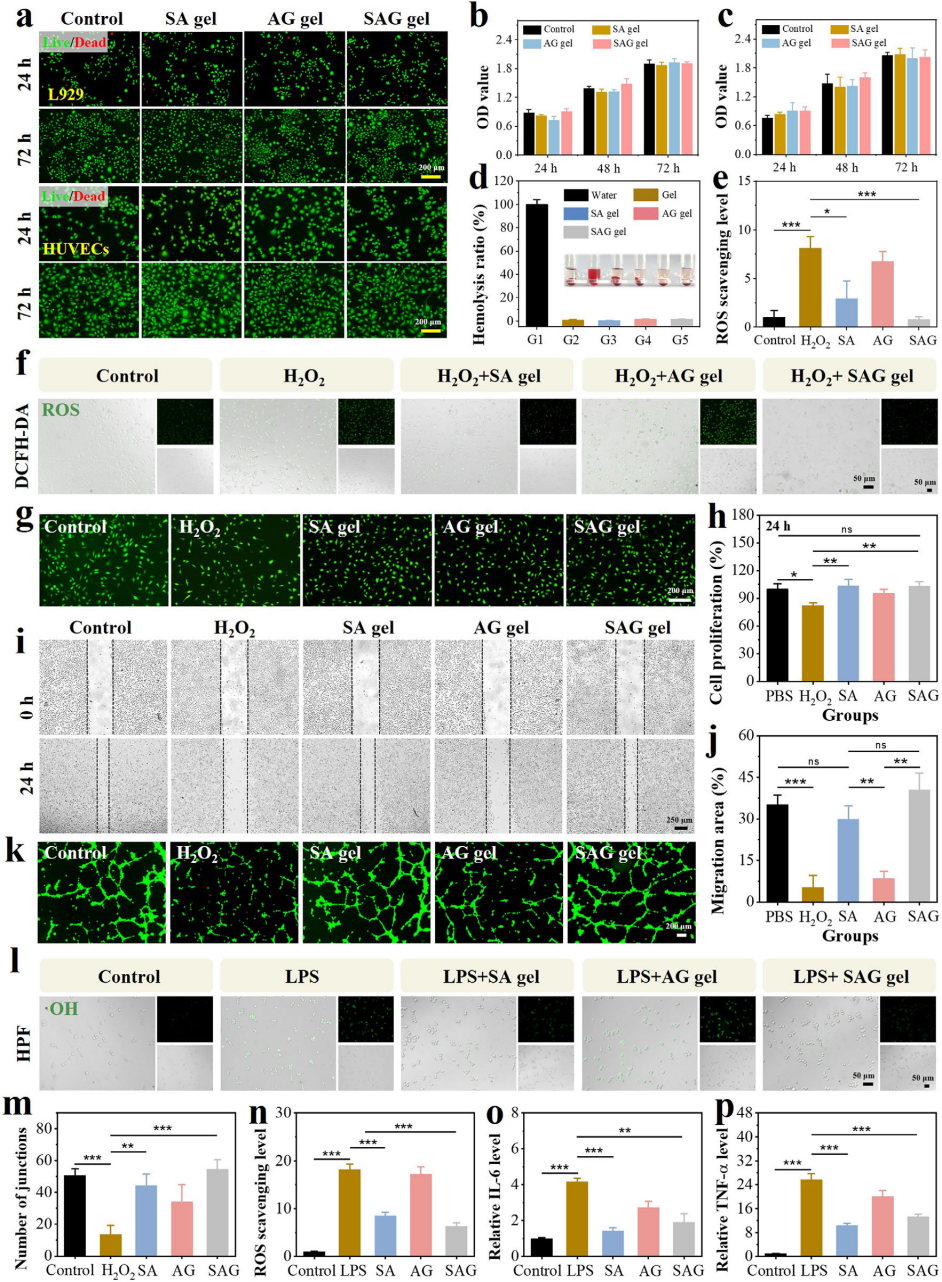
(a) Biocompatibility evaluation of SA gel, AG gel, and SAG gel by using L929 cells and HUVECs. (b) The viability of L929 cells following incubation with SA gel, AG gel, and SAG gel for different days. (c) The viability of HUVECs following incubation with SA gel, AG gel, and SAG gel for different days. (d) Hemocompatibility evaluation of SA gel, AG gel, and SAG gel. (e, f) Intracellular ROS scavenging ability of SAG gel measured by ROS assay kit. Data represent mean ± SD (n = 3). ^*^ *p* < 0.05; ^***^ *p* < 0.001. (g, h) Proliferation evaluation of H_2_O_2_‐treated L929 cells following incubation with different gels. Data represent mean ± SD (n = 3). ^*^ *p* < 0.05; ^**^ *p* < 0.01; ns, not significant. (i, j) Migration capacity of H_2_O_2_‐treated L929 cells after different treatments. Data represent mean ± SD (n = 3). ^**^ *p* < 0.01; ^***^ *p* < 0.001; ns, not significant. (k, m) Tube formation ability and quantitative junctions of H_2_O_2_‐treated HUVECs after being incubated with different gels. Data represent mean ± SD (n = 3). ^**^ *p* < 0.01; ^***^ *p* < 0.001. (l, n) •OH scavenging performance of LPS‐treated RAW 264.7 cells after received treatment of SAG gel. Data represent mean ± SD (n = 3). ^***^ *p* < 0.001. (o, p) Effects of SAG gel on the IL‐6 and TNF‐α secretion from LPS‐stimulated RAW 264.7 cells. Data represent mean ± SD (n = 3). ^**^ *p* < 0.01; ^***^ *p* < 0.001.

When biomaterials are used in vivo, the biocompatibility is a critical characteristic that needs to be evaluated. The biological activity of the generated SAG hydrogels was first evaluated at the cellular level. Live/Dead staining results in Figure 4a demonstrated that L929 cells and HUVECs maintained high cell activity after incubation with SAG hydrogel extracts for 3 days. Besides, CCK‐8 results also revealed that L929 cells and HUVECs that received the treatment of SAG hydrogel extracts showed an obvious proliferation trend after 3 days of culture, of which was similar to that of PBS group (Figure 4b,c). Furthermore, the hemolysis rates in Figure 4d for all hydrogels were found to be below 5%, demonstrating the good hemocompatibility. Benefiting from the superior ability of SA to decompose ROS, the ability of SAG hydrogel to scavenge the excessive ROS within HUVECs was investigated. After the HUVECs were incubated with the H_2_O_2_ additive, the intracellular ROS contents were detected by using a commercial ROS indicator (DCFH‐DA) (Figure 4e,f). HUVECs treated with SAG hydrogels demonstrated obviously weaker green fluorescence than the cells treated by H_2_O_2_ and AG hydrogels, suggesting that the SAG hydrogels can effectively eliminate the excess ROS in HUVECs. Based on the fact that RAW 264.7 cells are commonly used immune cells, the ROS in RAW 264.7 cells was also evaluated. The SAG hydrogel treatment can also significantly relieve the ROS levels in RAW 264.7 cells (Figure S7). Furthermore, owing to the ability to eliminate ROS of SAG hydrogels, it is anticipated that such hydrogel demonstrated the obvious facilitated effect on the cell growth and proliferation. To demonstrate this, the cell proliferation after ROS treatment was evaluated (Figure 4g,h, and Figure S8). L929 cells cultured with H_2_O_2_ treatment can result in cell death, while SA treatment enabled the higher cell viability compared with that in H_2_O_2_ group after 24 h. Notably, the SAG group also showed an obvious cell proliferation rate in contrast to H_2_O_2_ group, and there was no difference in the cell proliferation treated with PBS and SAG hydrogel after 24 and 48 h, suggestive of the protection ability of SAG hydrogels against ROS. To verify the effect of SAG hydrogels on re‐epithelialization processes of chronic wounds, the scratch test was carried out (Figure 4i,j). Compared with the no‐treatment control group, a H_2_O_2_ environment can significantly inhibit the migration of L929 cells. Notably, culturing L929 cells under a H_2_O_2_ condition and subsequently incubating with SAG hydrogel significantly increased the migration rate of L929 cells, suggesting that the SAG hydrogel could eliminate the superfluous ROS to relieve oxidative stress of cells, and further contribute to re‐epithelialization, thus facilitating chronic wound healing. Furthermore, vascularization also plays a crucial role during wound healing. Based on this, tube formation assay was performed to assess angiogenic capacity (Figure 4k,m). Compared with the control group, HUVECs cultured under ROS environment can significantly hinder tube formation. Whereas, for ROS‐incubated HUVECs, the SAG gel group showed a significant increase in tubular branches relative to the H_2_O_2_ and AG group, revealing that the SAG hydrogel positively contributed to the remodeling of oxidative stress homeostasis in HUVECs. Collectively, these results demonstrated that the SAG hydrogel can significantly reduce oxidative stress in cells, which in turn promotes diverse cellular functions, such as proliferation, migration, and angiogenesis under complex chronic wound microenvironment.

Excessive ROS can either oxidize biomolecules or bioactive proteins so as to trigger signaling cascades that cause the onset and progression of inflammatory diseases [47, 48]. Among the various ROS, •OH serves an important role in the biological oxidation‐reduction system, whereas its overproduction may break the balance and induce an inflammatory response [49, 50]. Thus, to demonstrate the anti‐inflammatory ability of SAG hydrogel, RAW 264.7 cells were stimulated by lipopolysaccharide (LPS) to produce a superfluous amount of •OH under severe oxidative stress, as demonstrated by the increased intracellular fluorescence detected by the hydroxyphenyl fluorescein (HPF) probe (Figure 4l,n). Upon incubation with SA hydrogels, the fluorescence became weakened, suggesting its excellent •OH scavenging capability. Nevertheless, the fluorescence of cells in AG group displayed negligible changes compared to the LPS group. Notably, the fluorescence of cells showed a significant decrease in the SAG group, signifying the robust free radical scavenging efficacy. Apart from the direct induction of body damage, ROS can also lead to organ injury through the production of excess proinflammatory cytokines [51, 52]. Thus, we studied the effect of SAG hydrogel on ROS‐induced inflammatory response by using RAW 264.7 cells. After treatment with LPS, the levels of interleukin‐6 (IL‐6) and tumor necrosis factor‐alpha (TNF‐α) were sharply increased (Figure 4o,p). In contrast, upon treatment with SA and SAG hydrogels, the cytokine levels can be obviously reduced when compared with that in LPS group, indicative of their potent anti‐inflammatory activity. Collectively, the SAG hydrogels displayed broad therapeutic benefits for inflammatory‐related disease by eliminating ROS and reducing proinflammatory cytokines, contributing to the chronic inflammatory wound healing.

**FIGURE 5:**
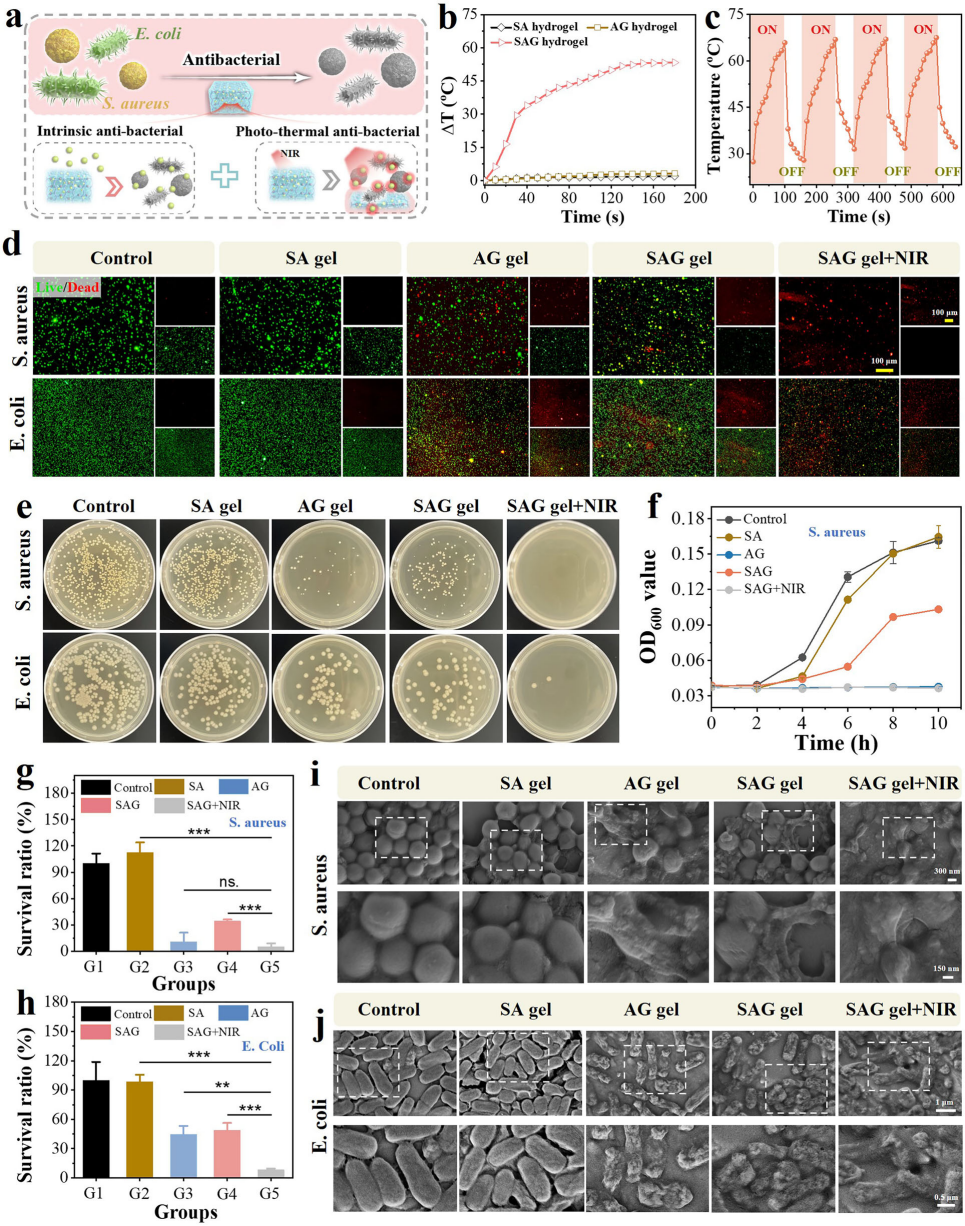
(a) The antibacterial mechanism of the SAG hydrogel. (b) Temperature changes of the SA hydrogel, AG hydrogel, and SAG hydrogel under NIR laser. (c) Temperature changes of the SAG hydrogel after the cyclic cooling‐heating process. (d) Live/Dead staining of *S. aureus* and *E. coli* after different treatments. (e) Photos of bacterial colonies of *S. aureus* and *E. coli* after receiving the different treatments. (f) The growth curve of *S. aureus* after different treatments. (g, h) The bacterial survival ratio after treated differently. Data represent mean ± SD (n = 3). ^**^ *p* < 0.01; ^***^ *p* < 0.001; ns, not significant. (i, j) SEM images and the corresponding magnified images of *S. aureus* and *E. coli* after treated differently.

Given the in situ formation of inorganic Ag NPs within the SAG hydrogel, it is anticipated that the hydrogel possesses good photothermal conversion abilities. To investigate the photothermal performances of SAG hydrogels, an 808 nm laser was applied on the hydrogel. The temperature elevation capabilities of SA hydrogel, Ag hydrogel, and SAG hydrogel under the same laser density were evaluated. As shown in Figure 5b, SAG hydrogel displayed a higher temperature elevation under NIR irradiation (2 W/cm^2^), whereas SA and Ag hydrogel showed slight temperature changes, suggesting that the SAG hydrogel possessed an outstanding photothermal conversion capacity. In addition, varying the laser power densities from 0.5 to 2 W/cm^2^, the temperatures of SAG hydrogels showed an increased trend (Figure S9). Moreover, the insignificant decrease in the maximum temperature was observed over four cycles of irradiation, suggesting its outstanding photothermal recycling stability (Figure 5c). Together, these results revealed that the formed SAG hydrogels show excellent photothermal performance.

Based on the excellent photothermal properties of in situ formed Ag NPs, the synergistic antibacterial abilities of SAG hydrogels were investigated against *Escherichia coli* (*E. coli*) and *Staphylococcus aureus* (*S. aureus*) (Figure 5a). First, Live/Dead bacterial staining was carried out to assess the antibacterial effect. As shown in Figure 5d, a small amount of dead bacteria with red fluorescence of propidium iodide (PI) was observed in the control group. Conversely, the SAG gel+NIR treatment, when irradiated to reach a temperature of approximately 50 °C, produced significantly stronger red fluorescence than that observed in the AG gel and SAG gel groups, indicating the potent antibacterial ability of such hydrogel. The temperature of 50 °C was applied for sterilization because the fact that 50°C displayed negligible damage to the wound tissue but high efficiency on antimicrobial infection [53]. The intensified antibacterial property was ascribed to the robust photothermal capacities of SAG gel with the assistance of in situ formation of Ag NPs. Although the antibacterial of SAG hydrogel was weaker than that of AG hydrogel owing to the Ag^+^ consumption during the reaction process with SA, the enhanced biosafety can be achieved through the transformation of AG hydrogels into SAG hydrogels. Additionally, an agar plate counting test was conducted to investigate the antibacterial ability of SAG hydrogel (Figure 5e). In contrast to control and SA gel group, the colony numbers of *S. aureus* and *E. coli* co‐incubated with SAG hydrogel were significantly reduced under 808 nm NIR laser irradiation. Although the antibacterial ability in SAG hydrogel was reduced relative to AG hydrogel, obviously decreased colony numbers were still be observed in SAG gel group when compared to the other groups.

Moreover, the quantitative bacterial killing ratio of SAG hydrogel was assessed (Figure 5g,h). Following NIR laser irradiation for 10 min, the SAG hydrogel can achieve nearly complete eradication (≈100%) of *E. coli* and *S. aureus*. In contrast, the bactericidal efficiency of the SAG hydrogel without NIR irradiation was about 66% for *S. aureus* and 52% for *E. coli*, respectively. These results demonstrated that SAG hydrogel with NIR irradiation displayed outstanding antibacterial performance. Also, after different treatments, the OD value at 600 nm of bacterial suspensions were recorded with time to evaluate bacterial activity (Figure 5f and Figure S10). Both AG and SAG gel+NIR treatment showed an obvious influence on bacterial growth compared with the other groups. Although the SAG hydrogel also displayed effective sterilization behavior at the first hours, such suppression effect can be disabled with the prolonged time. Bacterial growth was prominently inhibited in the SAG group with NIR laser irradiation, which can be attributed to the photothermal effect of SAG hydrogels. Furthermore, bacterial morphologies after different treatments were observed via SEM (Figure 5i,j). Compared with the bacteria in control and SA gel group observed smooth and intact surface membrane, obvious contraction, membrane damage, and serious distortion accompanied with apparent leakage were observed for the bacteria in AG gel group and SAG+NIR gel group. The result can be attributed to the biocidal Ag NPs and photothermal antibacterial activity of SAG hydrogels, leading to the damage of the integrity of bacteria membrane and ultimately causing the bacterial death. Together, all these results manifested that the SAG hydrogel had effective bactericidal activity for treating the infected chronic wounds.

**FIGURE 6:**
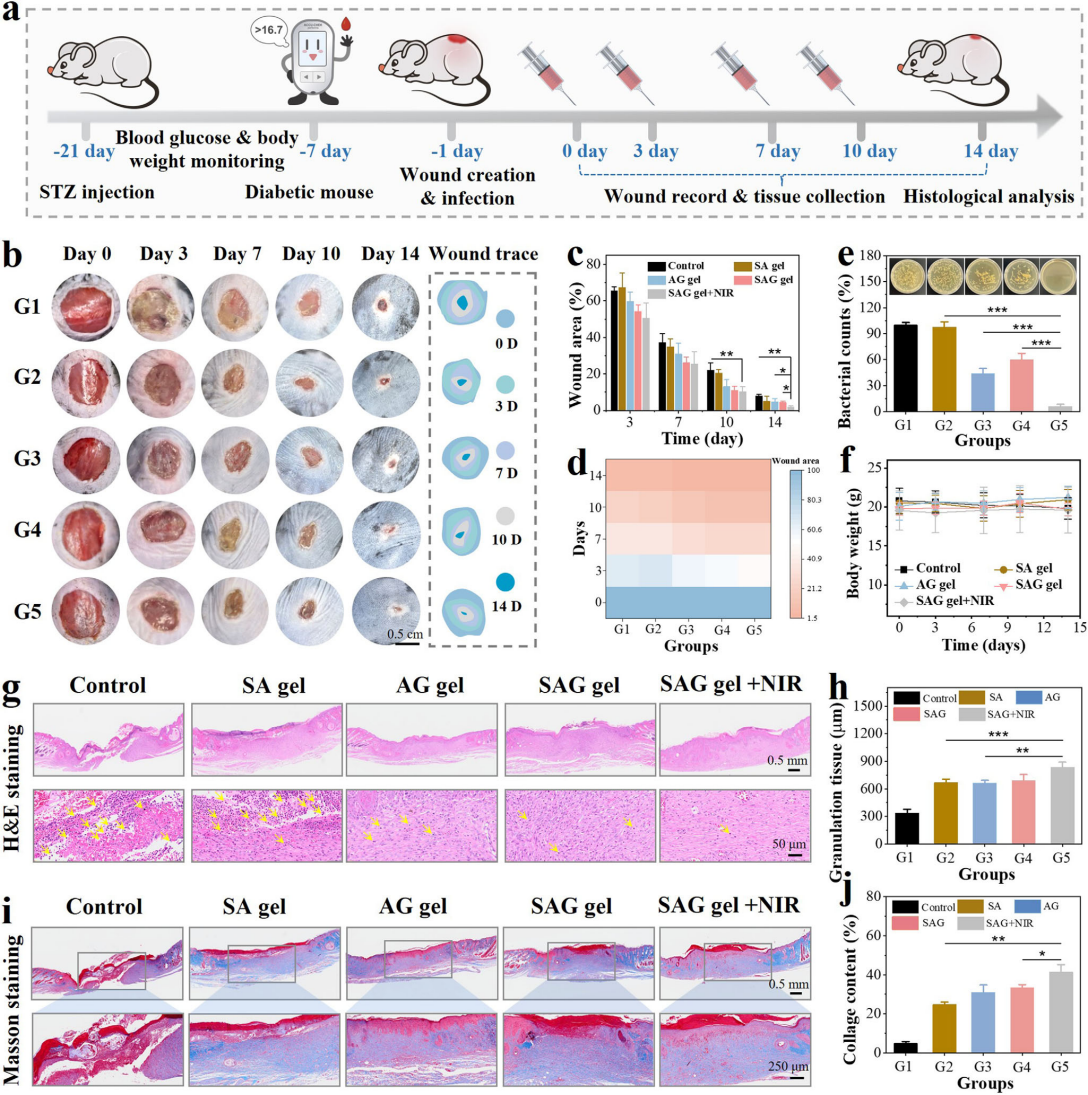
(a) Schematic illustration of the in vivo experimental timeline for evaluating the therapeutic performance of the SAG hydrogel. (b) Representative photographs of wounds in the different treated groups. (c, d) Wound area analysis over time for groups after different treatments. Data represent mean ± SD (n = 5). ^*^ *p* < 0.05; ^**^ *p* < 0.01. (e) Quantitative *S. aureus* colonies collected from different wounds after 3 days' treatment. The inserted images show the *S. aureus* colonies from wounds at day 3 cultured on agar plates. Data represent mean ± SD (n = 3). ^***^ *p *< 0.001. (f) The body weight changes of mice after different treatments over time. (g, h) H&E staining and the width of granulation tissues from wound tissues collected from mice after 14 days. Yellow arrows indicated the inflammatory cells at the wound bed. Data represent mean ± SD (n = 3). ^**^ *p* < 0.01; ^***^ *p* < 0.001. (i, j) Masson staining and the collagen contents of different wound tissues collected from mice after 14 days of treatments. Data represent mean ± SD (n = 3). ^*^ *p *< 0.05; ^**^ *p* < 0.01.

The SAG hydrogel displays robust adaptability, rapid self‐healing, dual antimicrobial, and enhanced antioxidant properties, all of which are expected to be effective for infected chronic wound healing. Thus, the therapeutic efficacy of SAG hydrogels for infected chronic wounds in the diabetic mouse model was evaluated. The schematic diagram illustrates the preparation of infected chronic wounds, and the related treatment and observation (Figure 6a). Specifically, the diabetic mouse model was first established in C57BL/6J mice through injecting streptozotocin (STZ) and the mice with blood glucose levels above 16.7 mm were confirmed diabetic mice. Subsequently, wounds with a diameter of 1 cm were created on the mouse dorsum with the infection of *S. aureus*, followed by treatment with G1 (Control), G2 (SA gel), G3 (AG gel), G4 (SAG gel), and G5 (SAG gel+NIR). In the SAG gel+NIR group, an 808 nm NIR laser (1 W/cm^2^) was applied on the wounds for 10 min. At the scheduled time points, the hydrogel dressings at each wound site were removed, and the wound images were recorded. Macroscopically, wounds in the SAG gel+NIR group healed faster than the other groups, with only about 1.8% of the initial wound area remaining on day 14 (Figure 6b–d). To quantitatively assess the antimicrobial efficiency of the SAG hydrogel, after wounds being infected for 3 days, the bacteria at the wound beds were gathered and reinoculated on agar plates (Figure 6e). Fewer bacterial colonies in the SAG gel+NIR group were observed than that in the other groups, and the bacteria survival rate was reduced to 6.0%, indicating that the SAG hydrogel presented effective antibacterial performance under NIR laser irradiation. This result was also in accordance with the in vitro antimicrobial test results. Throughout the treatment period and healing progress, a small weight fluctuation was observed in SAG gel+NIR group, demonstrating the slight systemic toxicity of SAG gel+NIR treatment (Figure 6f).

To thoroughly evaluate the therapeutic efficiency of different treatments, wounds were collected at day 14 for hematoxylin and eosin (H&E) and Masson's trichrome staining. As illustrated in Figure 6g,h, the skin defects in the SAG gel+NIR group were almost closed, along with an intact epithelium, several skin appendages (e.g., hair follicles and sebaceous glands), and almost completely regenerated dermal tissues. Besides, a significantly lower number of inflammatory cells was found in the wound bed after SAG gel+NIR treatment compared with the other treatment groups. As a comparison, the incomplete epithelialization with abundant inflammatory cell infiltration in control group was observed. In addition, the thickness of granulation tissues in SAG gel+NIR group was greatly larger than AG and SA group, suggesting the faster regeneration process. The rapid wound healing can be due to the enhanced antioxidant, dual antimicrobial abilities, and significant anti‐inflammatory performance of SAG hydrogels with NIR laser irradiation. Masson staining indicated that the control group possessed only a small number of loose collagen fibers (Figure 6i,j). In contrast, wounds treated by SAG gel+NIR displayed enhanced collagen deposition as well as denser collagen fibers relative to the other groups, demonstrating the better wound healing outcome. Systemic toxicity evaluation indicated that the SAG hydrogels showed no damage to any of the major organs after 2 weeks of treatment (Figure S11). Also, serological test results of blood urea nitrogen (BUN), alanine aminotransferase (ALT), and aspartate aminotransferase (AST) further demonstrated the excellent biocompatibility of SAG hydrogels (Figure S12). Additionally, the toluidine blue staining was performed to evaluate mast cells in wound tissues after different treatments (Figure S13). The SAG hydrogel treatment group showed the few numbers of mast cells similar to that of the control group, indicating negligible allergic reactions. Furthermore, given the significant role of immunoglobulin E (IgE) in allergic reactions, blood IgE levels following different treatments were measured to evaluate the potential allergenicity of the SAG hydrogel (Figure S14). The IgE concentration in the SAG group was similar to that observed in the other groups, further suggesting that the SAG hydrogel does not induce allergic responses. Because the current study focused on a treatment period of 14 days, and the SAG hydrogel is designed as a temporary wound dressing, a long‑term biosafety assessment was not necessary for the intended application. Together, these results clearly suggested that SAG gel+NIR treatment remarkably facilitated granulation tissue formation and collagen deposition, by which the tissue reconstruction of diabetic wounds was facilitated.

**FIGURE 7:**
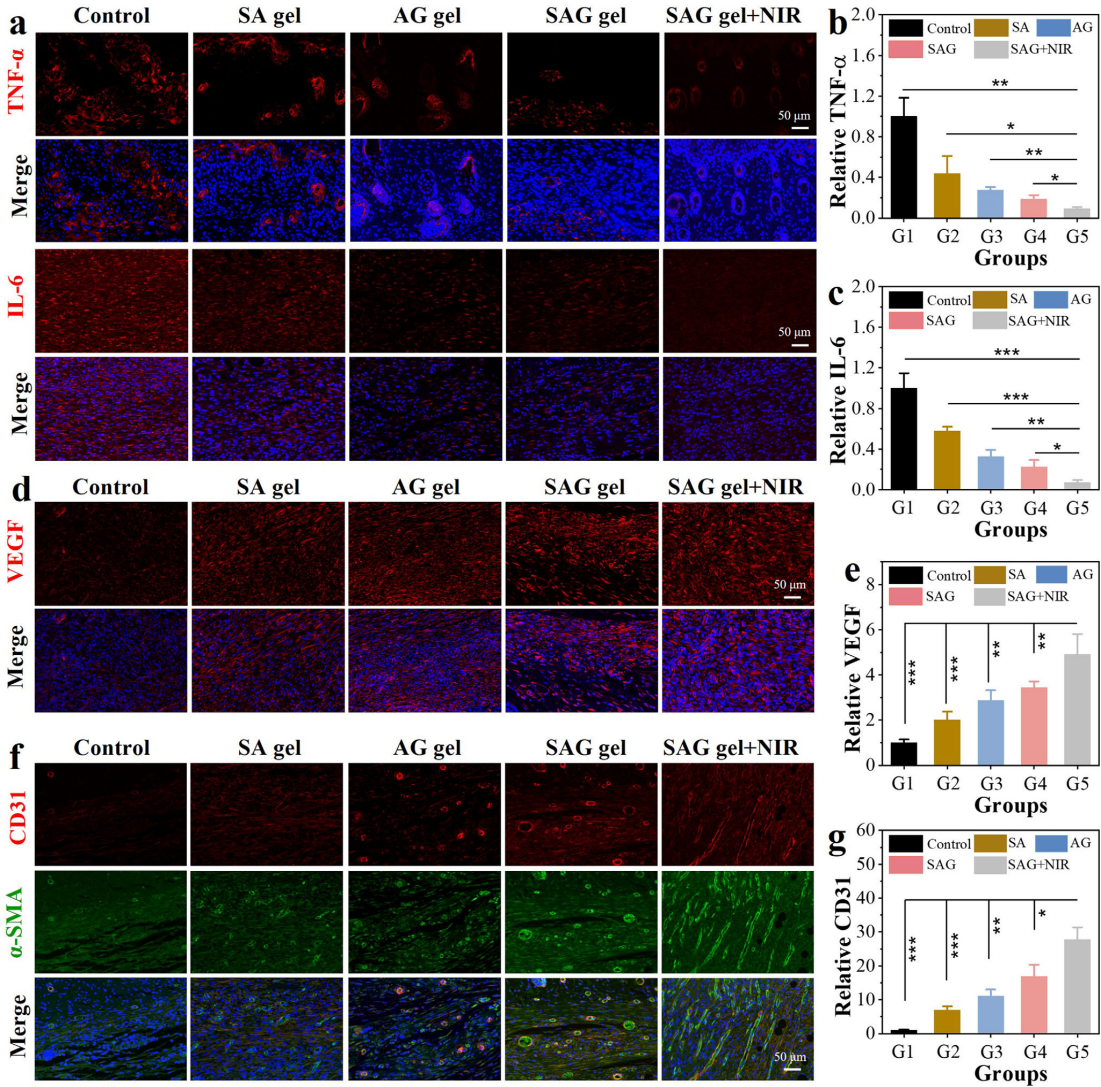
(a–c) Fluorescence staining and the corresponding semiquantitative analysis of TNF‐α and IL‐6 for each group after 14 days. Data represent mean ± SD (n = 3). ^*^ *p* < 0.05; ^**^ *p* < 0.01; ^***^ *p* < 0.001. (d, e) Fluorescence images of VEGF, and its corresponding semiquantitative analysis. Data represent mean ± SD (n = 3). ^**^ *p* < 0.01; ^***^ *p* < 0.001. (f, g) Fluorescence images of α‐SMA and CD31, and their corresponding semiquantitative analysis of CD31. Data represent mean ± SD (n = 3). ^*^ *p* < 0.05; ^**^ *p* < 0.01; ^***^ *p* < 0.001.

The persistent inflammatory response is the primary obstacle that seriously disturbed the wound repair process. In order to further evaluate the anti‐inflammatory ability and pro‐healing mechanism of SAG hydrogels, immunofluorescence staining analyses for TNF‐α and IL‐6 within the wounds were performed on day 14 (Figure 7a–c). The SAG gel group exhibited the weaker fluorescence compared to the SA gel and AG gel group, while SAG gel+NIR treatment induced the lowest expression of TNF‐α and IL‐6. Such improved anti‐inflammatory effects of SAG gel with NIR laser can be attributed to the dual anti‐bacterial behavior of SAG hydrogel under NIR and its excellent anti‐oxidation performance. Vascular endothelial growth factor (VEGF) is a key marker to indicate the blood vascular tissue regeneration. Thus, to demonstrate the effect of the SAG gel on the vascularization, the immunofluorescence staining of VEGF at the wound sites was performed in Figure 7d,e. The SAG gel+NIR treated groups displayed higher VEGF expression levels than the other groups after 14 days, indicating that the SAG gel+NIR treatment can improve vascularization compared to the other groups, which is likely due to the SAG gel with NIR could significantly alleviate the inflammation response. Furthermore, to understand the underlying wound healing mechanism of SAG gel on re‐epithelialization, the vascularization and cell proliferation at wound sites were evaluated. As depicted in Figure 7f,g, the relative fluorescence intensity of α‐SMA and CD31 in SAG gel+NIR group was more pronounced than that in the other groups, and the levels of CD31 after SAG gel+NIR treatment were about 4 and 2.5 times higher than that of the SA gel and AG gel group, respectively, indicating that SAG gel with NIR could more effectively facilitate angiogenesis. Together, we can conclude that the SAG gel effectively facilitates the wound healing process by reducing inflammation and promoting VEGF, α‐SMA, and CD31 expression in the wound beds. The enhanced in vivo wound repair may be attributed to the synergistic effects of efficient antibacterial, excellent ROS scavenging ability, improved anti‐inflammatory effect, enhanced cell proliferation and migration, and promoted angiogenesis. Although the antibacterial efficacy of the SAG hydrogel was lower than that of the AG hydrogel, its comprehensive therapeutic effects are better than AG hydrogels owing to the reduced cytotoxicity and its ability to facilitate the transition from the inflammatory to the proliferative phase. Consequently, the SAG hydrogel demonstrates considerable potential for use as wound dressings in clinical practice.

## 3 Conclusion

In summary, this work developed an integrative immunoregulatory hydrogel for the accelerated infected diabetic wound healing by the rapid wound closure, antioxidative, and antibacterial ability. The hydrogels were gelled rapidly upon mixing the benzaldehyde and cyanoacetate groups‐modified dextran, AgNO_3_, and SA, during which the in situ self‐mineralization of Ag NPs occurred within the hydrogel. The resulting hydrogel was endowed with the ROS‐scavenging, antibacterial, and the immunoregulatory capabilities. When used as wound dressings for infected chronic wounds, the hydrogels can achieve rapid wound closure, oxidative stress alleviation, bacterial eradication, and regulation of inflammatory responses for accelerated wound healing. Collectively, this versatile strategy, combining in situ gelation with self‐mineralization, offers a promising therapeutic platform not only for infected diabetic wounds but also for broader applications in chronic disease‐related tissue engineering.

In comparison with some reported Ag NPs‐incorporated hydrogels for treatment of infected wounds, the SA‐accelerated gelling hydrogel with rapid self‐mineralizing capacity in this work offers several distinct merits. First, the SA‐catalyzed dynamic hydrogels achieve the fast solidification, which not only prevents the precursor solution leakage but also endows the hydrogel with ROS‐scavenging capability, thus ultimately benefiting the immediate wound coverage and enhancing multiple cellular functions in the wound bed. Besides, although various Ag NPs‐based hydrogels have been developed, the majority of them rely on the incorporation of pre‐synthesized Ag NPs into the hydrogel matrix. This approach may introduce impurities due to the complex synthesis process of Ag NPs, potentially leading to the systemic cytotoxicity. Moreover, possible aggregation of the pre‐synthesized Ag NPs can compromise their photothermal efficiency and result in weak antibacterial performance. In contrast, our self‐mineralized hydrogels generate Ag NPs directly within the matrix during gelation process, ensuring uniform distribution and stable immobilization, providing a green and high‐efficiency platform for in situ Ag NPs formation under mild conditions. In this system, SA serves as a catalyst to accelerate gelation, as a mineralization accelerator to mineralize Ag NPs within the hydrogel, and as an antioxidant for biological functions. Thus, this design maximizes photothermal efficiency of the Ag NPs, greatly enhances antibacterial effects, reduces systemic toxicity, and simultaneously endows the hydrogel with ROS‐scavenging ability. The advantages of our system over conventional Ag NPs‑based hydrogels were summarized in Table S1. The proposed in situ biomineralization strategy offers a novel strategy for developing next‐generation multifunctional hydrogel wound dressings for complex tissue injuries.

## 4 Experimental Section

### 4.1 Materials and Cells

Dextran (*M*_n_ = 70 000 g mol^−1^) was obtained from Aladdin. 4‐Formylbenzoic acid and cyanoacetic acid were purchased from Alfa Aesar. Sodium ascorbate (SA) was purchased from Aladdin. AgNO_3_ (0.5 g/L) was from Macklin. *E. coli* and *S. aureus* were obtained from Hopebio Bio‐Technology Co., Ltd. 1‐Ethyl‐3‐(3‐dimethylaminopropyl) carbodiimide and 4‐dimethylaminopyridine were bought from Energy Chemical. All the chemicals were reagent grade and used as obtained. The L929 cells (RRID: CVCL_0462Best), HUVECs (RRID: CVCL_2959), and RAW 264.7 cells (RRID: CVCL_0493) were obtained from BioChannel Biological Technology Co., Ltd. and iCell Bioscience (Shanghai, China), respectively. The CCK‐8 and live/dead cell kit were obtained from Thermo Fisher Scientific. SYTO and propidium iodide were bought from Keygen Biotech.

### 4.2 Characterizations

Fluorescent images were gained using an inverted fluorescence microscope (ZEISS Axio Vert. A1, Germany). Rheology tests were carried out on an Anton Poar Physica MCR 301 rheometer. The surface morphology of hydrogels was examined with a scanning electron microscope (ZEISS GeminiSEM 360). Absorption spectra were obtained with a UV–vis–NIR spectrophotometer (CARY5000, USA).

### 4.3 Preparation and Characterization of SAG Hydrogels

Benzaldehyde‐modified dextran (17 mg) and cyanoacetate‐modified dextran (16.5 mg) were dissolved in 300 µL AgNO_3_ solutions (AgNO_3_ concentration: 0.1 g/L) at a 1:1 molar ratio of cyanoacetate‐to‐benzaldehyde group. SA with different contents was subsequently added into this mixture and the SAG hydrogel can be formed immediately. As a control, the SA hydrogel without addition of AgNO_3_ and the AG hydrogel without using SA were separately prepared following the aforementioned procedure. Gelation times of the hydrogels were determined as the time when the sample was not flowing within 30 s after inverting the vial.

### 4.4 Rheological Analysis of SAG Hydrogels

Mechanical properties of hydrogels were confirmed via an Anton Poar Physica MCR 301 rheometer. For all the rheology measurements, the hydrogel precursors or as‐prepared hydrogel samples were kept on the plate and measured immediately under different conditions. Besides, a layer of silicon oil was employed for sealing the hydrogel edge to prevent water from evaporating. First, time sweep test was carried out to confirm the gelation time of hydrogels with different SA contents. The gelation time was identified from the intersecting point of G′ and G′′ (strain: 1%, frequency: 1 rad/s). Next, a frequency sweep test of the SAG hydrogel was conducted with a range of frequency from 0.1 to 100 rad/s, from which the mechanical strength at varying frequencies can be verified. Then, shear strain sweep of SAG hydrogel was also carried out to confirm the critical breaking strain. Based on the obtained critical value at the intersecting point of G′ and G′′, the self‐healing capability for SAG hydrogels can be confirmed using an oscillatory step‐strain test. In brief, such hydrogel was alternately subjected to a high strain (800%) to disrupt network and a low strain (1%) to allow for recovery. To further illustrate the self‐healing capacity of SAG hydrogels, four pieces of SAG hydrogel dyed with red and green, respectively, were put together and incubated at room temperature for nearly 20 min. These hydrogel pieces were tightly connected to each other and hardly disrupted when being lifted.

### 4.5 ROS Free Radical Scavenging Capacity In Vitro

For DPPH• scavenging assay, different hydrogels and SAG hydrogel with different contents were mixed with 500 µL DPPH ethanol solution (0.3 mm), and reacted for 30 min followed by measuring the absorbance at 517 nm.

For PTIO scavenging assay, the PTIO solution (0.05 mg/mL) was first dispersed in PBS solution. Then, different hydrogels and SAG hydrogel with different contents were incubated with the PTIO solution (2 mL) for 2 h followed by measuring the absorbance at 557 nm.

For •OH scavenging assay, the ^•^OH was first prepared via a Fenton reaction based on Fe^2+^ (1 mm) and H_2_O_2_ (10 mm). Then, different hydrogels and SAG hydrogel with different contents were incubated with the mixed solution contained Fe^2+^ solution and 3,3′,5,5′‐tetramethyl‐benzidine (TMB, 10 mm) for 20 min. The ^•^OH concentrations could be confirmed by testing the intensity at 650 nm.

For ABTS scavenging assay, ABTS free radicals were first generated via mixing ABTS (0.2 mL, 7.4 mmol/L) and potassium persulfate solution (0.2 mL, 2.6 mmol/L) and reacting for 24 h. The obtained solution was diluted 50 times for further use. Subsequently, different hydrogels and SAG hydrogel with different contents were co‐incubated in ABTS solution for 10 min followed by measuring the absorbance at 734 nm.

### 4.6 Ag Release In Vitro

The SAG hydrogel (300 µL) was first prepared according to the above procedure, and then immersed into PBS solution (1 mL). At the schemed times, 0.5 mL of PBS solution was taken out, followed by the addition of fresh PBS solution (0.5 mL). The UV–vis–NIR spectrophotometer was applied to quantify the concentrations of released silver.

### 4.7 In Vitro Cytotoxicity of SAG Hydrogels

L929 and HUVEC cells (10^3^ cells/well) were used to evaluate the in vitro cytotoxicity. In brief, the cells were cultured for hours to achieve attachment, following the replacement of different hydrogel extracts (200 µL). At the predetermined time points, the CCK‐8 test was performed to confirm cell viability. Meanwhile, A Live/Dead assay was performed to visualize the cell morphologies after 1, 2, and 3 days of culture.

### 4.8 Hemocompatibility Assay

Red blood cell (RBC) suspensions were first obtained from mouse blood via multiple centrifugations and washing using PBS solution (pH 7.4). Then, the diluted RBC solution (5%, 500 µL) was incubated with SAG hydrogels (300 mg) for 1 h with the shaking rate of 100 rpm. Subsequently, the RBC solution was centrifuged and the supernatant absorbance (100 µL) was obtained by a microplate reader.

### 4.9 Photothermal Performance In Vitro

Photothermal behaviors for different hydrogels could be evaluated with an 808 NIR laser. Specifically, hydrogels were put under the 808 nm NIR laser for 3 min and the temperature was recorded in real‐time. Besides, by changing the laser power densities form 0.5–2 W/cm^2^, the effect of laser densities on the photothermal behaviors can also be confirmed. Furthermore, the photothermal stability of SAG hydrogels was investigated under the cyclic irradiation with an 808 nm laser (2.5 min on/2.5 min off). The cyclic process was repeated for 4 cycles.

### 4.10 In Vitro Antibacterial Experiments

First, different hydrogels were first prepared and underwent strict sterile treatment. Then, the bacterial (10^8^ CFU/mL) were treated by the following treatments: Control, SA gel, AG gel, SAG gel, and SAG gel+NIR, respectively for 4 h. In particularly, for the SAG gel+NIR group, the bacterial underwent NIR laser (1 W/cm^2^) for 10 min at the initial stage of the experiment. Subsequently, the bacterial resuspension (100 µL) was transferred into the 96‐well and OD value (600 nm) could be obtained. Furthermore, they were also diluted and added to the agar plate for another 12 h. The images of colony‐forming were obtained. In addition, for the bacterial after undergoing different treatments, the OD values at 600 nm were recorded over time to obtain the bacterial growth curves.

### 4.11 Intracellular ROS Scavenging Ability

For the assay, H_2_O_2_ was used as the positive control group. Briefly, HUVEC cells (10^4^ cells/well) in 96‐well plate were cultured overnight and then 10 µL H_2_O_2_ (final concentration: 100 µm) were added into each cell plate and incubated for 12 h. Subsequently, ROS‐treated cells were respectively co‐incubated with different hydrogels including the SA gel, AG gel, SAG gel, and SAG gel for 12 h. The contents of hydrogels applied in this experiment were fixed at 10 mg/well. After that, DCFH‐DA (10 µm) was added to each plate and the ROS contents in HUVECs were evaluated by detecting the fluorescence of the DCFH‐DA.

Among all the ROS, ·OH is the most representative one, which can cause the severe inflammatory reaction. Also, the LPS was used to increase the ROS level. Based on these, intracellular ·OH scavenging ability of the SAG hydrogel was evaluated as well. In brief, RAW 264.7 cells (10^4^ cells/well) in 96‐well plate were cultured and allowed to adhere overnight. Then, they were exposed to LPS (1 µg/mL) for 24 h following by the hydrogel treatments for another 24 h. Subsequently, intracellular ·OH levels were detected by fluorescent microscopy using the hydroxyphenyl fluorescein (HPF, 10 µm) probe.

### 4.12 Cell Proliferation Behavior

To confirm the cell proliferation behavior of cells under ROS environment, L929 cells (10^4^ cells/well) in 96‐well plate were first seeded with the addition of 100 µm H_2_O_2_ and incubated overnight. Then, different hydrogels (10 mg) were added into each plate and co‐cultured with ROS‐treated cells for different time. At the predetermined time, CCK‐8 solutions were added and OD value (450 nm) was measured. Meanwhile, Live/Dead test was performed to confirm cell proliferation behavior.

### 4.13 Cell Migration

To confirm the cell migration behavior of cells under ROS environment, L929 cells (3 × 10^5^ cells/well) in 6‐well plate were first seeded followed by the addition of 100 µm H_2_O_2_ and incubated 24 h. Next, a uniformly wide line was created in wells followed by adding different hydrogels. After co‐culture for another 24 h, the cell migration was recorded by using an optical microscope.

### 4.14 Tube Formation Assay

Tube formation of cells under ROS environment was studied. 200 µL of Matrigel was added to 48 well plates and H_2_O_2_‐treated HUVECs (10^5^ cells/well) were seeded on each surface of Matrigel for 4 h after the complete solidification of Matrigel. Then, these cells severally underwent the treatments of SA hydrogel, AG hydrogel, and SAG hydrogel with the same amounts for 6 h. The tube formation images were captured using microscope and the tube numbers were quantified with ImageJ software.

### 4.15 Anti‐Inflammatory Capacity

RAW 264.7 cells (10^5^ cells/well) were exposed to LPS (100 ng/mL) for 12 h after adhesion, followed by the treatment with the SA, AG, or SAG hydrogels for 24 h. After that, the cell supernatants were collected and tested for pro‐inflammatory cytokines of TNF‐α and IL‐6, and their concentrations can be confirmed using ELISA kit.

### 4.16 Diabetic Wound Healing Performance

Male C57BL/6J mice (5–6 weeks, 18–20 g each) were obtained from Beijing Vital River Laboratory Animal Technology Co., Ltd. Animal experiments were carried out according to the Laboratory Animal Care and Use Guidelines, and were approved by the Ethical Committee of Wenzhou Institute, University of Chinese Academy of Sciences (WIUCAS22080904). The therapeutic efficacy of SAG hydrogels on chronic wound repair can be assessed by using the *S. aureus*‐infected diabetic wound model. Such model was generated by intraperitoneal administration of STZ (50 mg/kg) into mice every other day, and mice with blood glucose contents over 16.7 mmol/L can be selected for further wound healing experiments. Specifically, after anesthesia was administered, a circular wound (diameter: 1 cm) was excised on the back skin followed by addition of *S. aureus* solution (10 µL, 10^8^ CFU). Then, these wounds were randomly assigned into 5 groups (n = 5), and different treatments were employed onto these wounds: (1) Control group, (2) SA hydrogel, (3) AG hydrogel, (4) SAG hydrogel, and (5) SAG hydrogel with NIR laser irradiation. All hydrogels were prepared using a mixture of 10% mixed functionalized dextran. The SA hydrogel contained an additional 1.5% SA, the AG hydrogel contained 0.01% AgNO_3_, and the SAG hydrogel contained both 1.5% SA and 0.01% AgNO_3_. For group (5), upon SAG hydrogel was sealed over the wounds, a NIR laser light (1 W/cm^2^) was applied on the wounds. At the scheduled time points, photographs of wounds were recorded and further analyzed using Image J software. At day 14, the skin around the wound site and the major organs were collected and then immersed in 4% paraformaldehyde and underwent H&E staining, Masson's trichrome staining, and immunofluorescence staining.

### 4.17 Statistical Analysis

All data in the experiment were performed with Origin 2023b and were reported as mean ± standard deviations (SD) (n≥3). The datasets were screened to identify and exclude outliers prior to analysis. Statistical evaluation was analyzed using Student's t‐test for comparison between two groups and two‐way analysis of variance (ANOVA) for multiple comparisons. The differences were considered statistically significant if ^*^ *p* < 0.05, ^**^ *p* < 0.01, or ^***^ *p* < 0.001.

## Author Contributions

Y. J. Zhao conceived the idea and designed the experiment; X. Y. Ding and W. Yang carried out the experiments. X. Y. Ding and G. F. Xu analyzed the data and wrote the manuscript. W. Z. Li contributed to the scientific discussion of the article.

## Conflicts of Interest

The authors declare no conflicts of interest.

## Supporting information




**Supporting File**: advs75881‐sup‐0001‐SuppMat.docx.

## Data Availability

The data that support the findings of this study are available from the corresponding author upon reasonable request.
