# Supplementary material for: Sodium Ascorbate‐Accelerated Gelling Hydrogels With Rapid Self‐Mineralized Capacity for Chronic Wounds
Source: Adv Sci (Weinh). 2026 May 29:e75881. Online ahead of print. doi: 10.1002/advs.75881 (PMC13335920; doi:10.1002/advs.75881)
Supplement: Supplementary file 1 — Supporting file: advs75881‐sup‐0001‐SuppMat.docx. [file ADVS-9999-e75881-s001.docx]

**Supplementary**

**Sodium ascorbate-accelerated gelling hydrogels with rapid self-mineralized capacity for chronic wounds**

Xiaoya Ding^1,*^, Wei Yang^1^, Wenzhao Li^2^, Guifang Xu^1,3,*^, Yuanjin Zhao^1,2,*^

^1^ MOE Innovation Center for Basic Research in Tumor Immunotherapy, Anhui Province Key Laboratory of Tumor Immune Microenvironment and Immunotherapy, The First Affiliated Hospital of Anhui Medical University, Hefei 230022, China

^2^ Department of Rheumatology and Immunology, Nanjing Drum Tower Hospital, School of Biological Science and Medical Engineering, Southeast University, Nanjing 210096, China

^3^ Department of Gastroenterology, The First Affiliated Hospital of Anhui Medical University,

Hefei 230032, China

Email: dingxiaoya@fy.ahmu.edu.cn; [xuguifang@fy.ahmu.edu.cn](mailto:xuguifang@fy.ahmu.edu.cn); yjzhao@seu.edu.cn


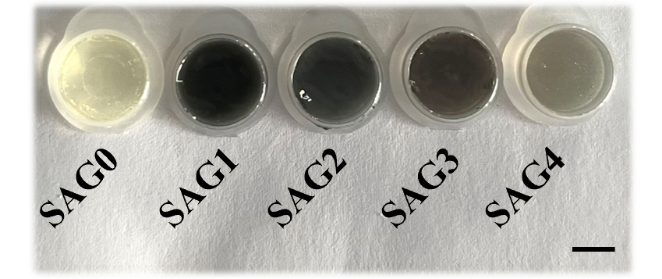


**Figure S1**. Photos of different SAG hydrogels. SAG0, SAG1, SAG2, SAG3, and SAG4 indicated the SAG hydrogels contained 0%, 0.05%, 0.025%, 0.01%, and 0.005% Ag^+^. Scale bar: 0.6 cm


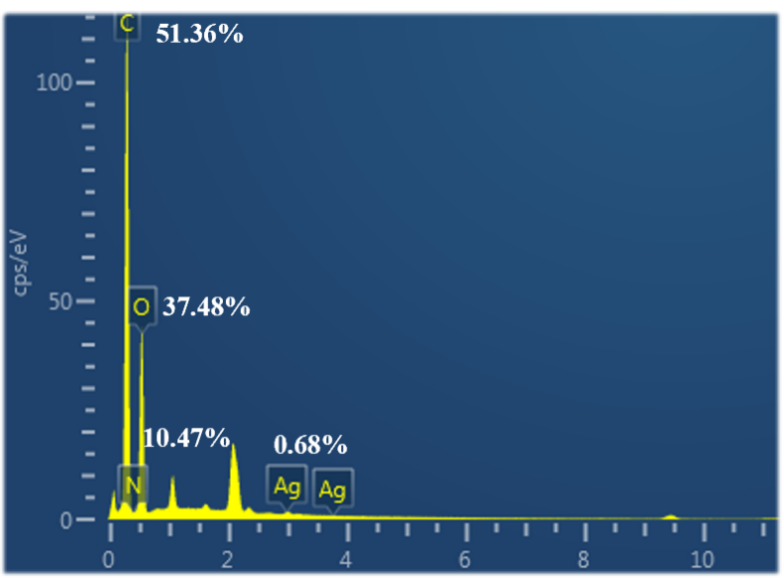


**Figure S2**. The EDS elemental profile of the SAG hydrogel.


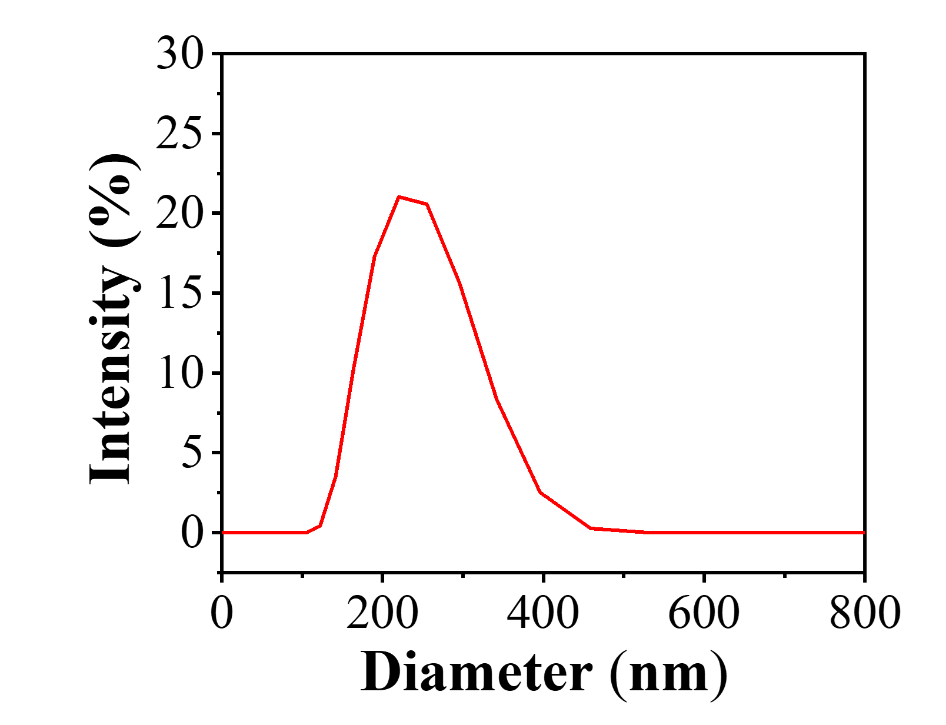


**Figure S3.** The average diameter of the formed Ag nanoparticles.


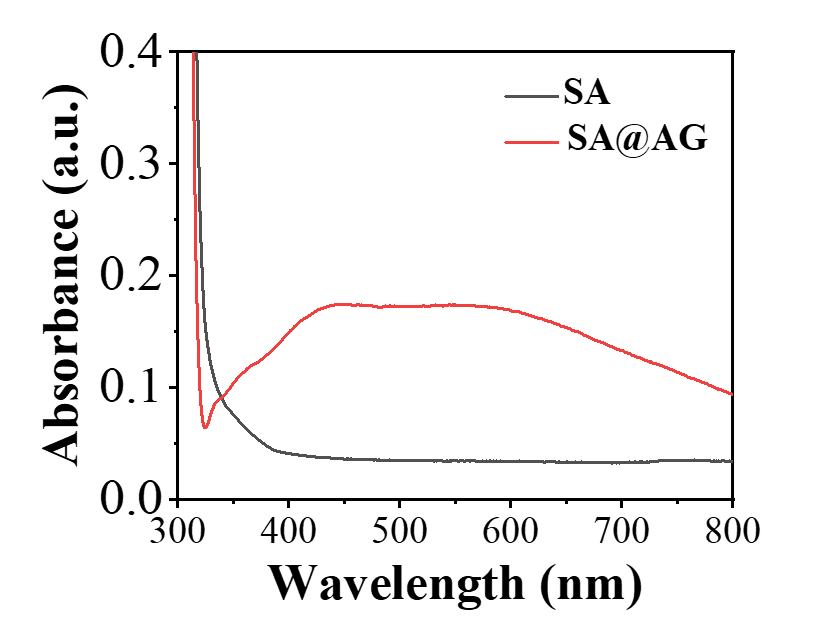


**Figure S4**. The absorbance of SA and SA@AG.


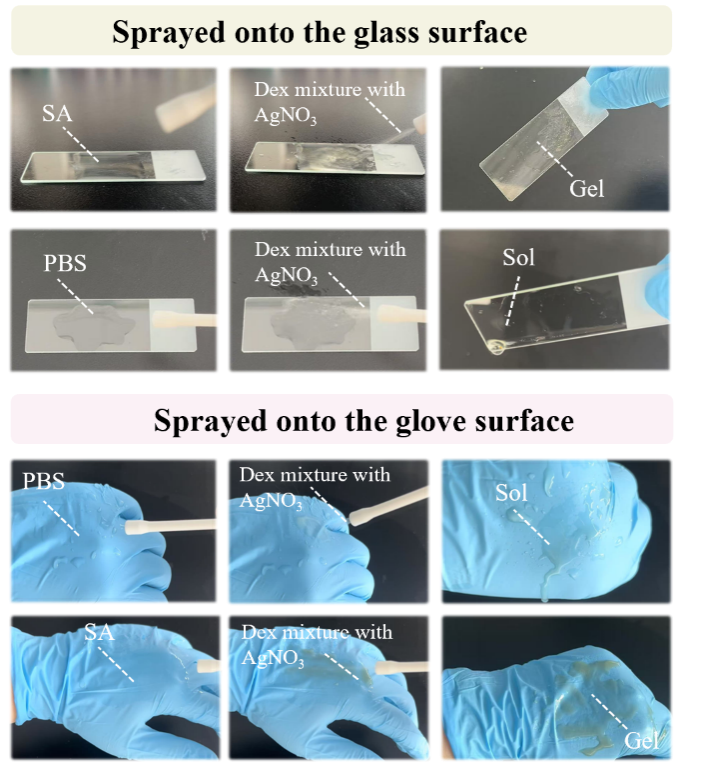


**Figure S5**. The hydrogel can be immediately formed when the precursor solutions were sprayed onto the surface of glass or gloves with a thin layer of SA solution.


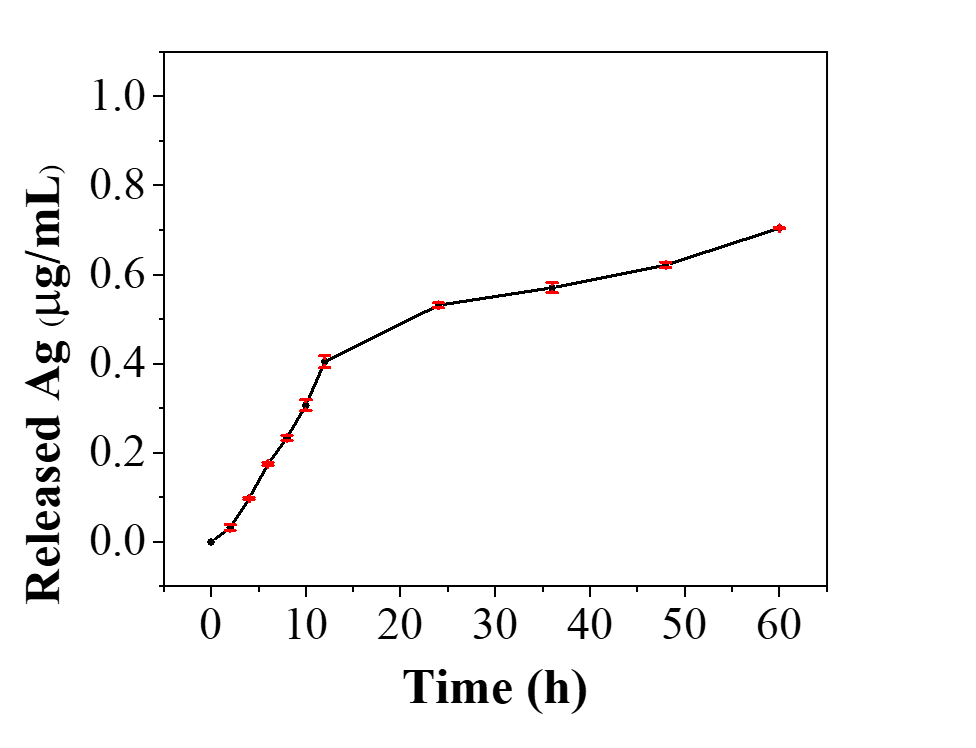


**Figure S6**. The release behavior of Ag NPs from SAG hydrogels. Data represent mean ± SD (n = 3).


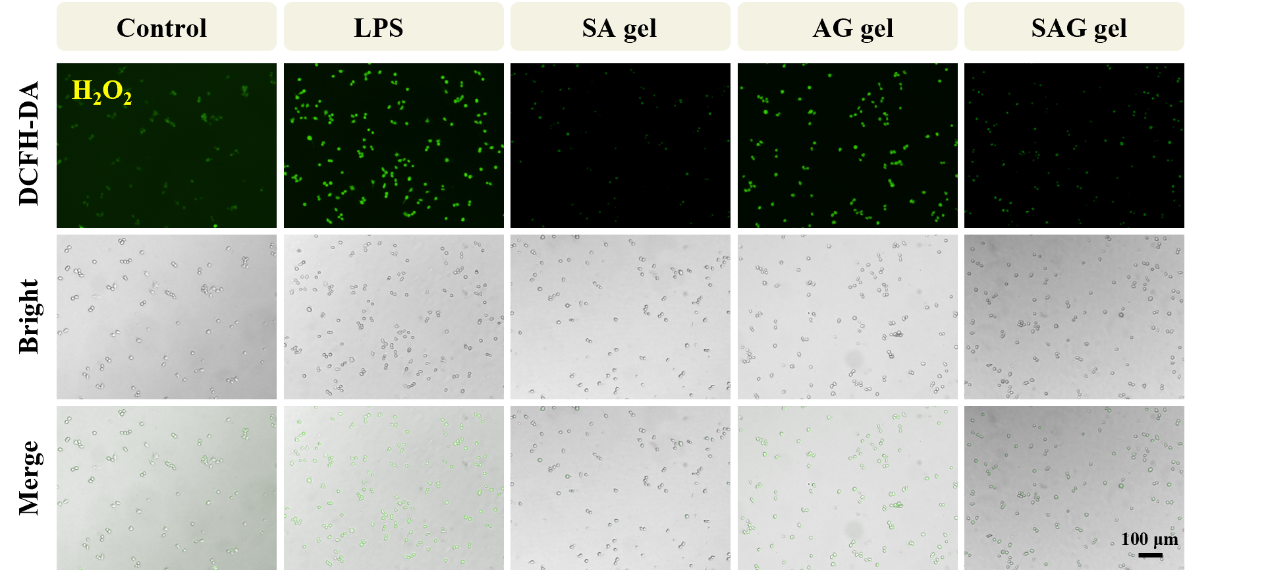


**Figure S7**. Intracellular ROS-scavenging capacity of SAG hydrogel on LPS-stimulated RAW 264.7 cells by using DCFH-DA fluorescent probe.


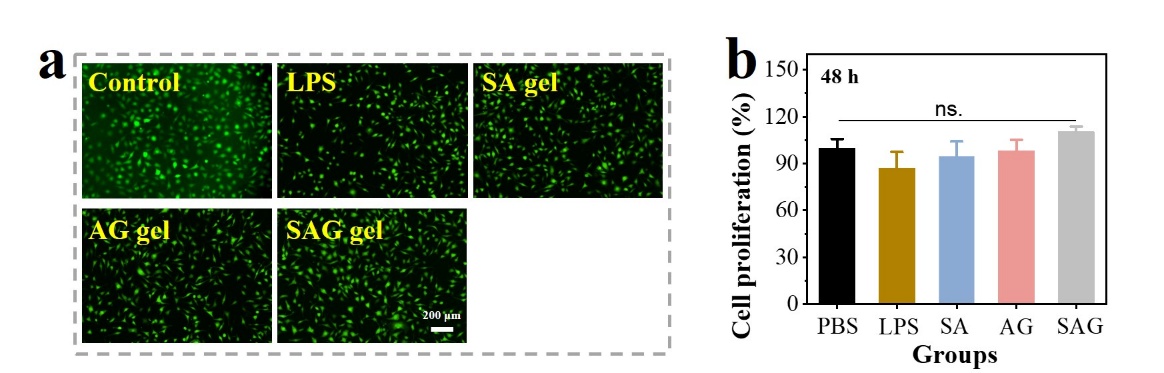


**Figure S8**. a) Live/Dead staining of L929 cells after incubation with SA gel, AG gel, and SAG gel for 48 h. b) Proliferation ratios of L929 cells after incubation with SA gel, AG gel, and SAG gel for 48 h.


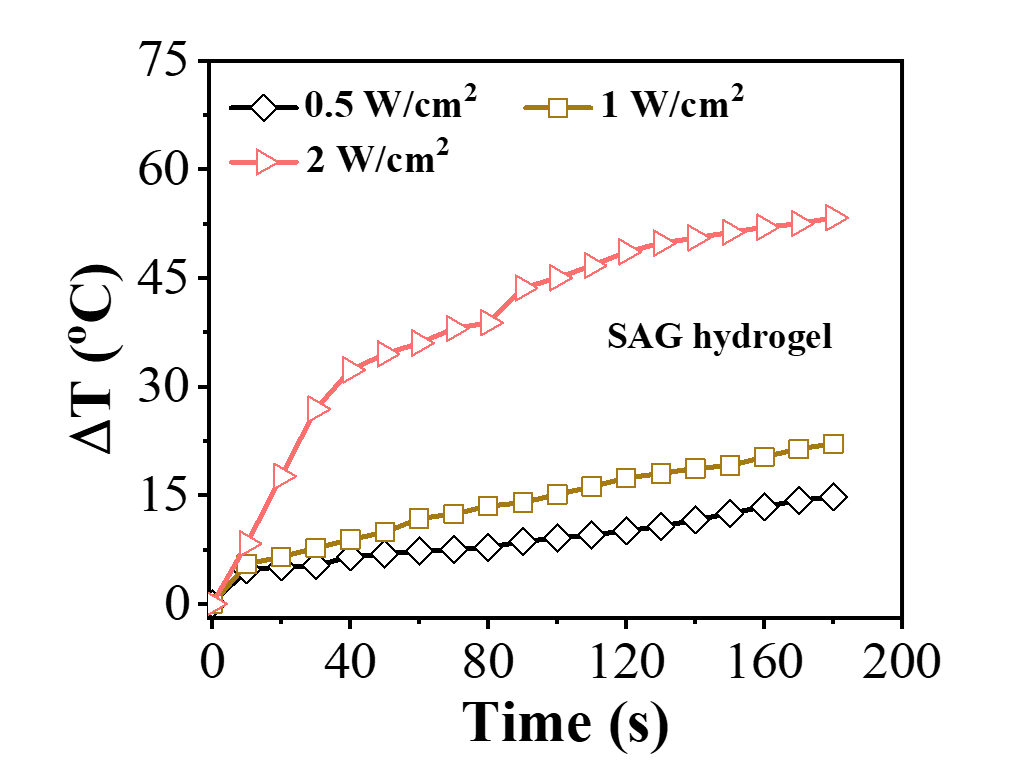


**Figure S9**. Temperature changes of SAG hydrogel under the NIR laser with different power densities.


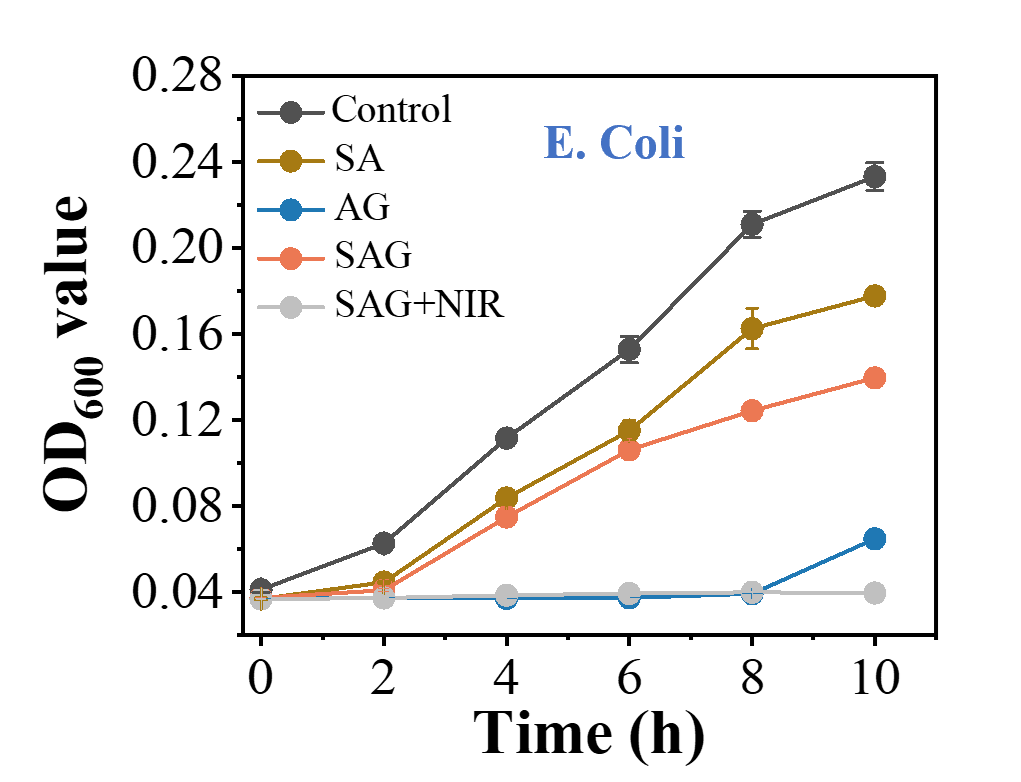


**Figure S10**. Growth curves of *E. Coli* after different treatments.


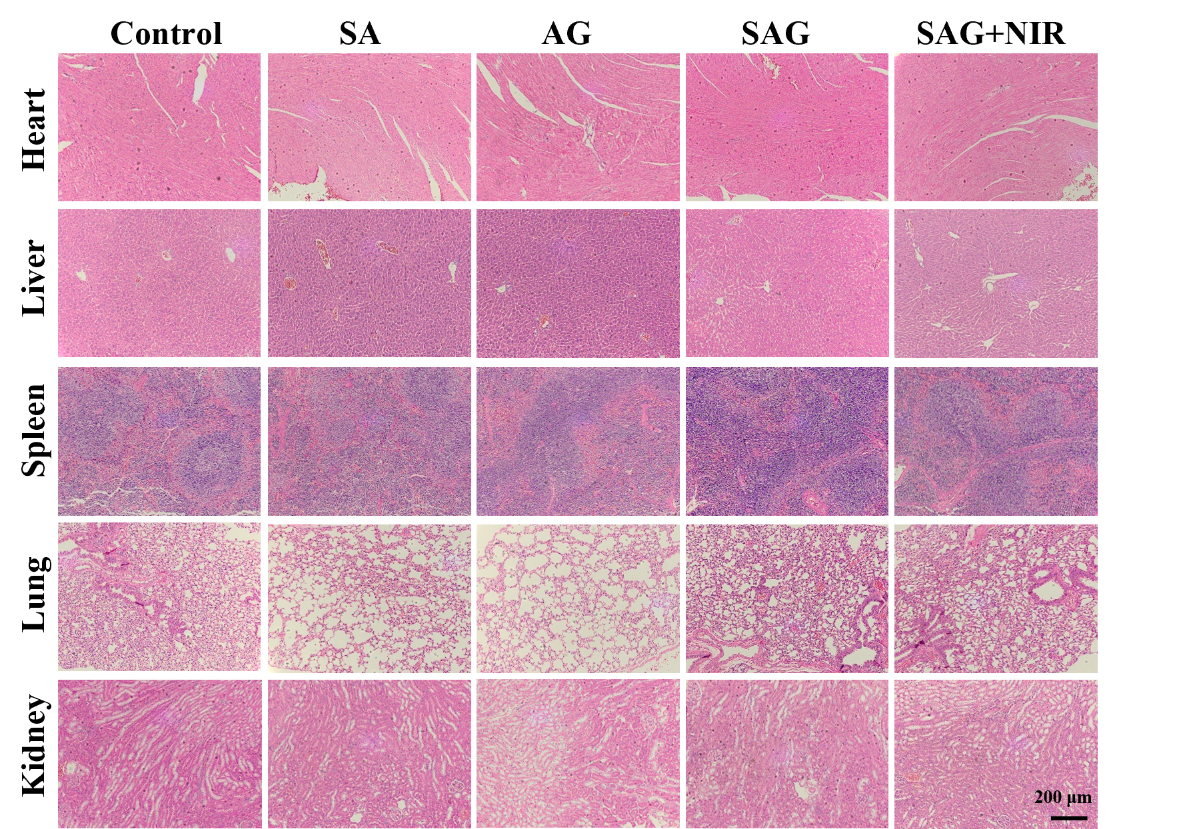


**Figure S11**. H&E staining of heart, liver, spleen, lung, and kidney from mice after treated differently at day 14.


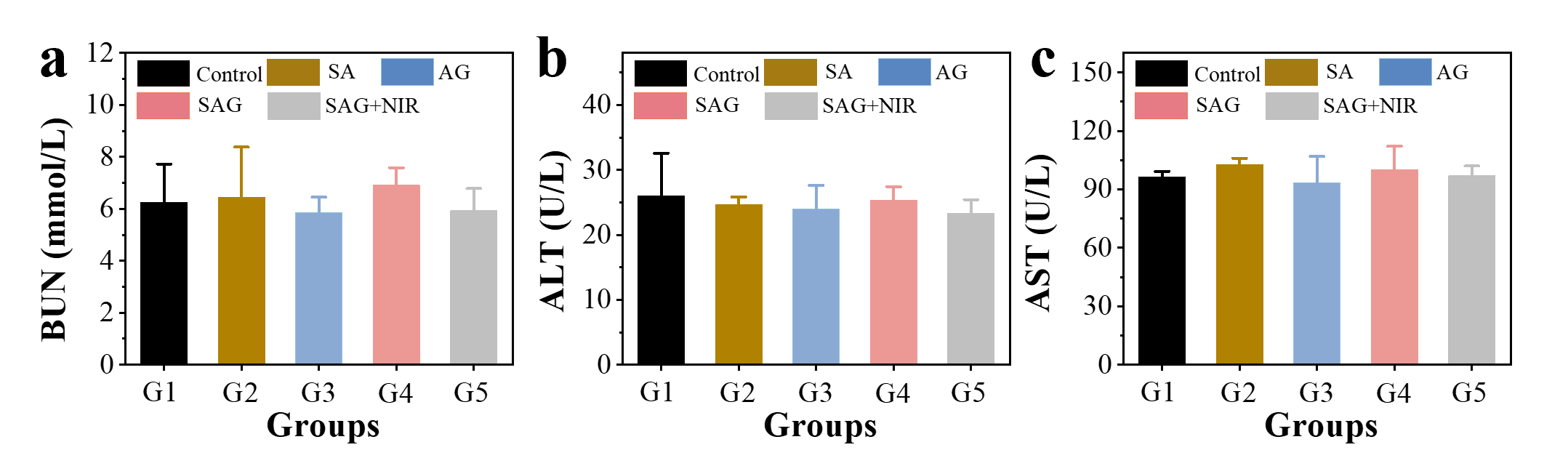


**Figure S12**. Biochemical parameters of liver function in serum containing a) BUN, b) ALT, and c) AST.


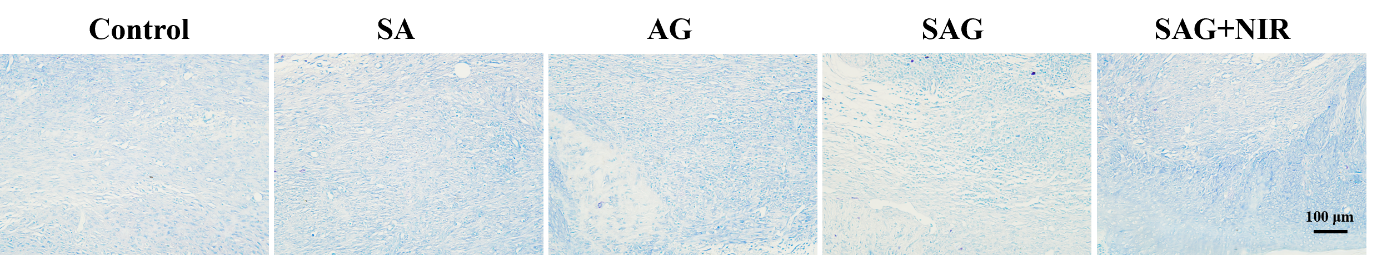


**Figure S13**. Toluidine blue staining of wound tissues from mice after different treatments.


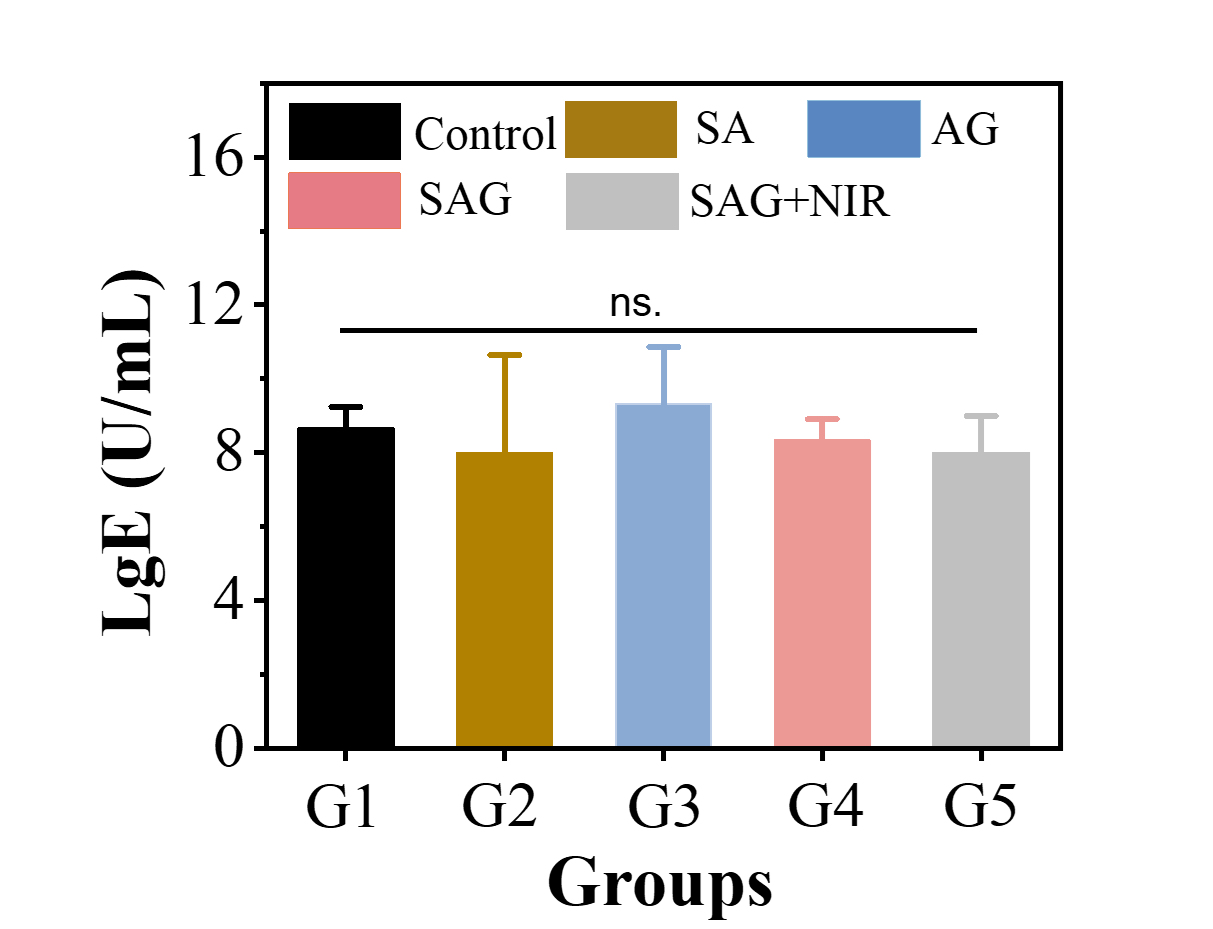


**Figure S14**. Immunoglobulin E (IgE) levels in the blood from mice after different treatments. Data represent mean ± SD (n = 3). ns, not significant.

**Table S1** Comparison of our work with the prior reports

| **Feature** | **Prior reports** | **This work** |
| --- | --- | --- |
| **Gelation mechanism** | Physical, slow chemical crosslinking or photo-crosslinking | SA-accelerated dynamic covalent crosslinking |
| **Gelation time** | Minutes to hours | < 30 seconds |
| **AgNPs incorporation** | Pre-synthesized and physically loaded | In situ self-mineralization (green, one-pot) |
| **Role of SA** | Not applicable | Gelation accelerator, reducer, antioxidant |
| **ROS scavenging** | Not present or indirect | Intrinsic and direct (from SA in network) |
| **Primary mechanism** | Single-function (antibacterial) | Spatiotemporal synergy (antibacterial, antioxidant, and immunomodulatory) |
| **Clinical potential** | Limited by slow gelation and complex preparation | High (ultrafast, ready-to-use, one-pot synthesis) |
